# Mechanosensitive TRPV4 channel guides maturation and organization of the bilayered mammary epithelium

**DOI:** 10.1038/s41598-024-57346-x

**Published:** 2024-03-21

**Authors:** Kärki Tytti, Koskimäki Sanna, Guenther Carla, Pirhonen Jonatan, Rajakylä Kaisa, Tojkander Sari

**Affiliations:** 1 Department of Applied Physics, School of Science, Aalto University, Espoo, Finland; 2 Faculty of Medicine and Health Technology, Tampere University, Tampere, Finland; 3 School of Social Services and Health Care, Tampere University of Applied Sciences, Tampere, Finland; 4 Tampere Institute for Advanced Study, Tampere University, Tampere, Finland

**Keywords:** TRPV4, Mechanosensing, Mammary epithelium, Differentiation, Breast cancer, Actin cytoskeleton, Epithelial integrity, Breast cancer, Mechanotransduction, Mechanisms of disease, Calcium signalling, Ion channel signalling

## Abstract

Biophysical cues from the cell microenvironment are detected by mechanosensitive components at the cell surface. Such machineries convert physical information into biochemical signaling cascades within cells, subsequently leading to various cellular responses in a stimulus-dependent manner. At the surface of extracellular environment and cell cytoplasm exist several ion channel families that are activated by mechanical signals to direct intracellular events. One of such channel is formed by transient receptor potential cation channel subfamily V member, TRPV4 that is known to act as a mechanosensor in wide variaty of tissues and control ion-influx in a spatio-temporal way. Here we report that TRPV4 is prominently expressed in the stem/progenitor cell populations of the mammary epithelium and seems important for the lineage-specific differentiation, consequently affecting mechanical features of the mature mammary epithelium. This was evident by the lack of several markers for mature myoepithelial and luminal epithelial cells in TRPV4-depleted cell lines. Interestingly, TRPV4 expression is controlled in a tension-dependent manner and it also impacts differentation process dependently on the stiffness of the microenvironment. Furthermore, such cells in a 3D compartment were disabled to maintain normal mammosphere structures and displayed abnormal lumen formation, size of the structures and disrupted cellular junctions. Mechanosensitive TRPV4 channel therefore act as critical player in the homeostasis of normal mammary epithelium through sensing the physical environment and guiding accordingly differentiation and structural organization of the bilayered mammary epithelium.

## Introduction

All tissues of the human body are exposed to various types of physical stresses, such as stretching, compression and alterations in the stiffness of the stroma. Such biophysical alterations are recognized by specific mechanosensitive cellular machineries that are able to read and transform physical information into altered activity of cellular signaling cascades^1,2^. Mechanosensitive machineries thus enable cells within the tissues to adopt their cellular structure and rigidity as well as direct cellular functions that are essential for the normal homeostasis of the tissues. During cancer progression, both stiffness and composition of the stroma undergo major alterations^3^. More commonly, the rigidity of the tissue increases and e.g., in breast cancers the stiffness of the mammary tissue can be even ten times higher than in normal tissue^4–7^. Such biophysical alterations can lead to abnormal activation of the mechanosensitive machineries and subsequently promote cellular changes linked to cancer progression. In addition, these mechanosensitive machineries themselves can become functionally defective during cancer progression, consequently interfering the ability of the cells to sense and respond to their biophysical environment.

One large category of cellular mechanosensors is composed by plasma membrane embedded ion channels within specific ion channel families^8–10^. Of these families, the transient receptor potential (TRP) family proteins form a large and conserved family of ion channel proteins that can recognize numerous extra-cellular cues and trigger specific downstream signaling cascades through spatio-temporal changes in the ion influx^8,11,12^. TRP family proteins can be further divided into seven subfamilies: TRPV (vanilloid), TRPA (ankyrin), TRPC (canonical), TRPM (melastatin), TRPML (mucolipin), TRPN (NOMPC), and TRPP (polycystin) families that contain altogether over 30 different cationic channel proteins^13^. Of these families, the subfamily of TRPVs (Transient receptor potential vanilloid-type cationic channels) has six members, TRPV1-6, of which TRPV4 is probably most studied in respect of its mechanosensitive features^14–19^. TRPV4 channel is widely expressed throughout different tissues but is especially abundant in epithelial tissues where it can regulate calcium influx in response to various stimuli from the extracellular environment ^12,19–23^. Ca^2+^ -influx, mediated by TRPV4, guides various physiological functions, including nociception through pressure-sensing and has a role in the development of functional vascular system^19,24,25^. Additionally, it plays a central role in cell volume regulation by acting as a membrane-stretch-sensing channel upon osmotic changes^26,27^. Besides these physiological functions, TRPV4 seems to be important for the maintenance of epithelial integrity through its role in the regulation of on adherens and tight junction proteins^28–33^. Further, TRPV4-mediated calcium influx seems to be essential for the integrity of cell–cell junctions in the skin keratinocytes^34,35^. In addition to its involvement in the control of cell adhesion proteins, TRPV4 can fine-tune organization of actin cytoskeleton and contractile features through changes in the calcium homeostasis at least in the cardiomyocytes, smooth muscle and vascular endothelial cells^25,36–39^.

TRPV4 levels are deregulated along the progression of several cancer types, including breast cancer^40,41^. TRPV4 expression seems to be highest in the basal-like subtype of breast carcinoma and its levels are also prominent in the metastatic lesions of invasive ductal carcinomas, correlating with tumor grade and size^42–44^. The mechanisms behind altered TRPV4 levels are poorly understood but at least stromal stiffening could play a role in this process due to the ability of TRPV4 to respond to the biophysical changes in its microenvironment, such as pressure and stiffness in some cell types^45–47^. Interestingly, TRPV4 itself has also been shown to modulate the biophysical microenvironment by controlling the expression of some extracellular matrix proteins^43^. The functional significance of high TRPV4 expression in breast cancers has been mainly linked to migration and spread of cancer cells^43,46,48–50^. TRPV4 has also been associated with angiogenetic process through the regulation of Rho kinase pathway in endothelial cells^48,49^ and may thus promote cancer cell spread through the induction of tumor vasculature. In addition, TRPV4 is linked to the invasive migration of cancer cells through the control of EMT (epithelial mesenchymal transition) markers and regulation of pathways upstream of actin cytoskeleton^50^. TRPV4-induced changes in actin-based structures therefore impact deformability and rigidity of the cancer cells, in this way favoring their potential to metastasize^42,43^.

In this study we wanted to investigate the role of mechanosensitive TRPV4 channel in the structural and functional homeostasis of the normal mammary epithelium. Mammary epithelium is bilayered, composed by an inner luminal epithelial (LE) and an outer basal, myoepithelial (ME) cell layer, and in 3D forming a branched tree-like network with ductal and lobular structures^51^. The development of the mature luminal and myoepithelial cells takes place in a strictly controlled, sequential manner via specific intermediate cell populations. The stem and progenitor cells with bipotent features are localized in the outer basal layer together with the contractile myoepithelial cells^51–53^. Here we show that TRPV4 is expressed in a tension-dependent manner and is found in all mammary epithelial cell types but especially prominent in the stem and progenitor cell populations. Depletion of TRPV4 led to differentiation bias of the stem-and progenitor cells with subsequent defects in the maturation of contractile myoepithelial cells. Furthermore, loss of TRPV4 was reflected to alterations in the signaling pathways upstream of actomyosin assembly and cellular forces exerted by the epithelial layers. These changes associated with the impaired ability of the mammary epithelial cells to maintain intact epithelial sheets and 3D morphology. All together, these data suggest that TRPV4 can act as a mechanosensor and guide maturation of the force producing cells within the mammary epithelium.

## Results

### TRPV4 is prominently expressed in basal stem and progenitor cell populations of the mammary epithelium

TRPV4 expression is deregulated in various cancers, including breast carcinomas. High TRPV4 protein levels are linked to basal-like breast cancer subtypes and its abnormal expression has also been found in the metastatic sites of invasive ductal carcinomas, linking TRPV4 to the mechanisms behind invasive potential^42–44,46,48–50,54^. While the role of TRPV4 in breast cancer progression has been assessed by various studies, its role in the homeostasis of normal mammary epithelium has gained less focus.

**Figure 1:**
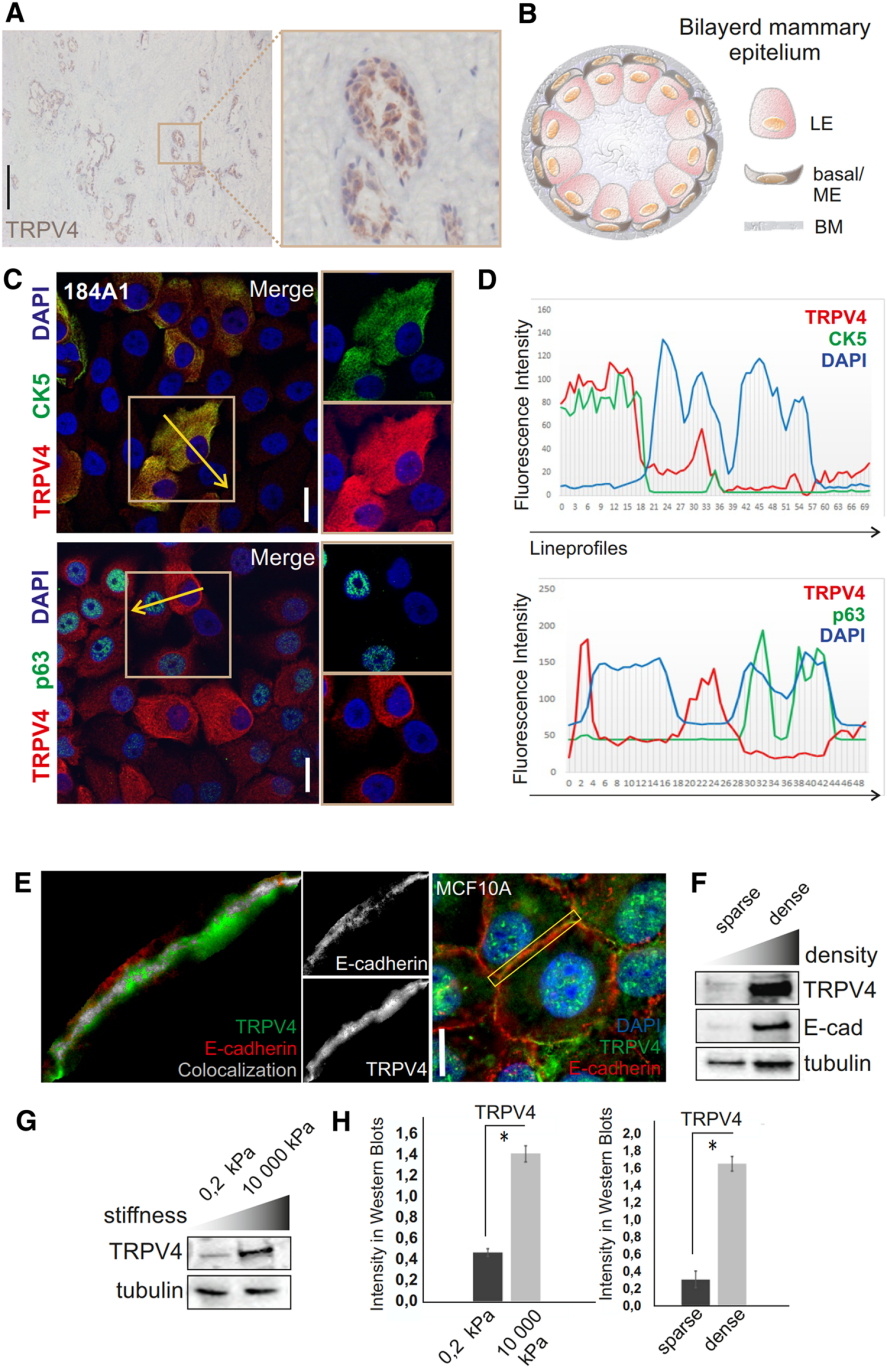
Expression of TRPV4 in the mammary epithelium. (**A**) IHC stainings of mammary tissue slices with TRPV4 antibody. Magnification of one bilayered epithelial structure is shown on the right side. Bar 200 um. **(B)** Illustration of the mammary epithelial structures and their distinct cell populations within the bilayered epithelium. Myoepithelial cells—ME cells; Luminal epithelial cells—LE cells; Basement membrane—BM. (**C)** Immunofluorescence stainings of 184A1 cells with TRPV4 antibody together with stem/progenitor marker CK5 or marker for mature ME cells, p63, revealed high expression of TRPV4 in the stem-and progenitor cell populations. TRPV4 indicated with red color and DAPI with blue. CK5 in the upper panel is indicated with green and p63 in the lower panel with green. Bar 20 um. (**D)** Lineprofiles of the indicated regions (yellow arrows within the marked boxes in (**C**) showing representative expression patterns of TRPV4 with either CK5 or p63. (**E)** Immunofluorescence stainings of confluent MCF10A cell cultures. Co-staining of confluent epithelial cell cultures with TRPV4 and E-cadherin shows concentration of TRPV4 at the cell–cell junctions together with E-cadherin. Colocalization analysed with Image J and indicated with the white color on the left side panel. E-cadherin-red; TRPV4-green; DAPI-blue. Bar 20 um. (**F)** TRPV4 expression is increased upon tension from cellular crowding as shown by the Western blots, performed from lysates done either from sparse or confluent MCF10A cultures. Besides TRPV4, E-cadherin levels were detected by specific antibodies. Tubulin acts as a loading control. (**G)** Stiffness-dependent expression of TRPV4 as shown by Western Blots from cellular lysates from 184A1 cells, cultured on 0.2 kPa substrates or plastic dishes (rigidity around 10 000 kPa). Tubulin acts a s a loading control. (**H)** Quantifications of the TRPV4 levels from Western Blots, related to (**F**,**G**). n for all samples is 3. Mean (+ /− SEM) is shown. *P < 0.05 (Paired t-test).

To assess how TRPV4 is expressed in the normal mammary epithelium and whether it is found in the contractile mammary epithelial basal layer, we utilized immunohistochemical, IHC, stainings of the mammary gland tissue slices as well as two normal human mammary epithelial cell lines, MCF10A and 184A1, containing a mixture of mammary epithelial cell populations^55–61^. IHC stainings of the human mammary tissue slices revealed a weak and diffuse staining in the mammary epithelial cells with no obvious concentration to either luminal or basal cell types (Figs. 1A,B, S1A). However, Western Blotting from cellular lysates, performed from isolated primary mammary epithelial basal cells, revealed high TRPV4 expression in this cell population (Fig. S1B). Additionally, immunofluorescence stainings of the mammary epithelial cell lines revealed that some cells within the mammary epithelial cell populations display higher TRPV4 expression and that TRPV4 is concentrated at the mature cell–cell junctions (S1C,D). To screen in more detail whether the high TRPV4 expression is concentrated in distinct epithelial cell populations, we did further stainings with cell-type specific markers. These studies revealed that highly TRPV4-expressing cells correspond to the stem-and/or progenitor cells, expressing Slug, CK5 and CK14 (Figs. 1C,D; S1E,F), while the expression was not so prominent in the mature, p63-positive, myoepithelial cells (Fig. 1C,D). Furthermore, TRPV4 expression levels were also found to be dependent on tension and cellular crowding: TRPV4 levels were clearly higher in cell cultures with high density and when cells were cultured on increasing stiffness (Fig. 1F–H). Together, these data indicate that TRPV4 is localized prominently in the stem/progenitor cells of the mammary basal layer and could be initial responder to mechanical changes in the microenvironment.

### Mechanosensitive TRV4 impacts lineage specific differentiation within the mammary epithelium

**Figure 2:**
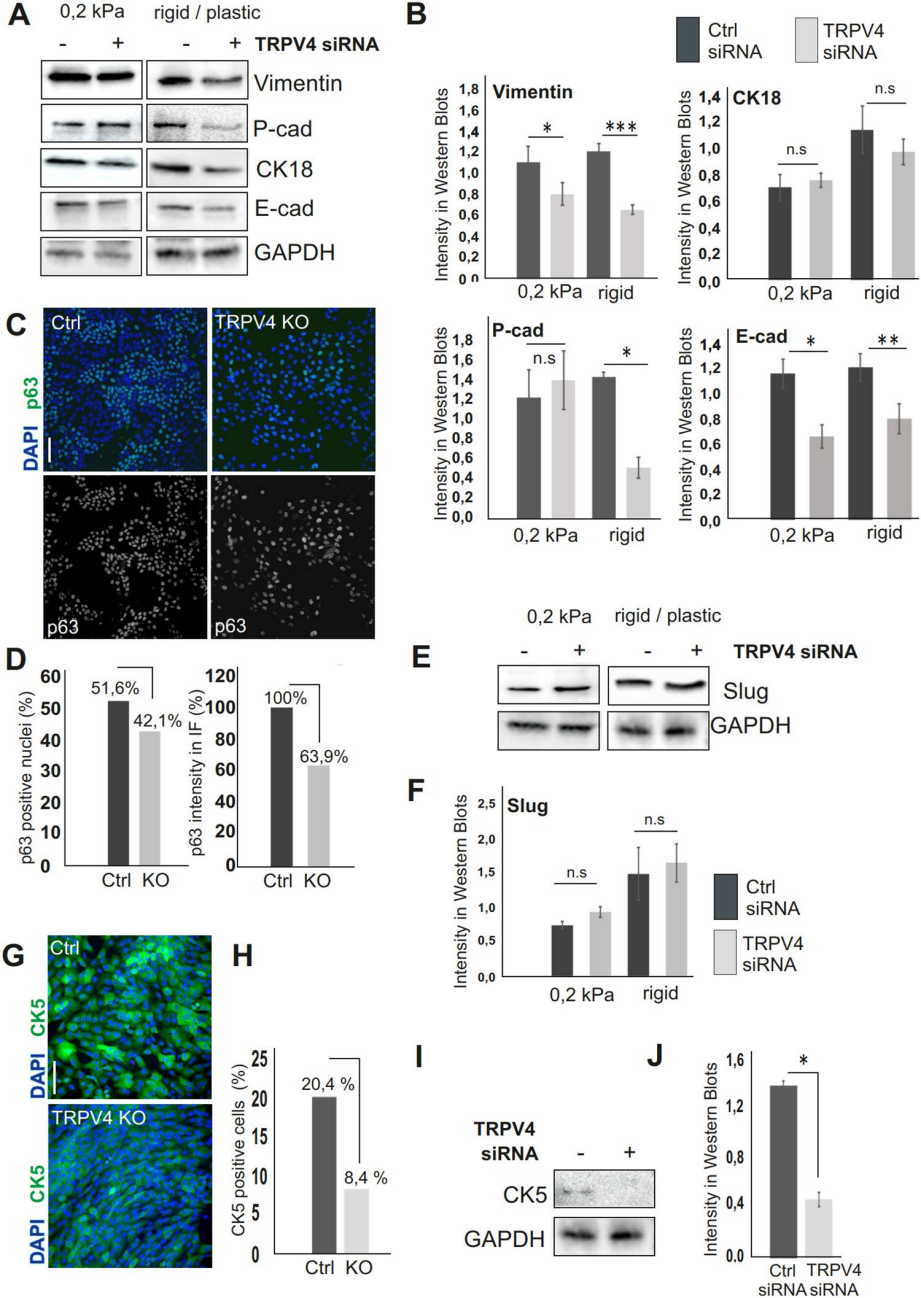
TRPV4 directs differentiation of the mammary epithelial cells. (**A**) 184A1 Mammary epithelial cells were treated with either ctrl siRNAs or TRPV4 targeting siRNAs, cultured on 0.2 kPa compliant matrices or on plastic. Cellular lysates were performed after 2 weeks of culture and both LE-and ME-specific markers were detected with Western Blotting. GAPDH was used as a loading control. (**B)** Quantification of the Western Blots, related to (**A**). n(Vimentin, ctrl, 0.2 kPa) = 6; n(Vimentin, siRNA, 0.2 kPa) = 6; n(Vimentin, ctrl, plastic) = 8; n(Vimentin, siRNA, plastic) = 8; n(P-cad, ctrl, 0.2 kPa) = 3; n(P-cad, siRNA, 0.2 kPa) = 3; n(P-cad, ctrl, plastic) = 3; n(P-cad, siRNA, plastic) = 3; n(CK18, ctrl, 0.2 kPa) = 9; n(CK18, siRNA, 0.2 kPa) = 8; n(CK18, ctrl, plastic) = 4; n(CK18, siRNA, plastic) = 4; n(E-cad, ctrl, 0.2 kPa) = 6; n(E-cad, siRNA, 0.2 kPa) = 6; n(E-cad, ctrl, plastic) = 7; n(E-cad, siRNA, plastic) = 7. Mean (+ /− SEM) is shown. *P < 0.05, **P < 0.01, ***P < 0.001, n.s = not significant (Paired t-test). (**C)** Myoepithelial marker, p63, was detected with IF stainings from ctrl MCF10A and TRPV4-depleted MCF10A cell lines. p63 shown with green color and dapi (nuclei) with blue. Bar 100 um. (**D)** Amount of p63 positive cells in percentage of the whole cell amount (dapi-stained nuclei) was calculated with Fiji by detecting p63 signal in the nuclei. Unspecific/low signal was avoided by using thresholding. Left side graph shows the amount of p63 positive cells (%) in ctrl and TRPV4 KO cell lines. The right side graph shows mean intensity of p63 signal in comparison to control cell signal (100%). (**E)** 184A1 Mammary epithelial cells were treated with either ctrl siRNAs or TRPV4 targeting siRNAs, cultured on 0.2 kPa compliant matrices or on plastic. Cellular lysates were performed after 2 weeks of culture and Slug was detected with Western Blotting. GAPDH was used as a loading control. (**F)** Quantifications of the Western Blots, related to (**E**). n(slug, ctrl, 0.2 kPa) = 6; n(slug, siRNA, 0.2 kPa) = 6; n(slug, ctrl, plastic) = 8; n(slug, siRNA, plastic) = 8. Mean (+ /− SEM) is shown, n.s = not significant (Paired t-test). (**G)** Stem/progenitor cell marker, CK5, was detected with IF stainings from ctrl MCF10A and TRPV4-depleted MCF10A cell lines. CK5 shown with green color and dapi (nuclei) with blue. Bar 100 um. **(H)** Amount of CK5 positive cells in percentage of the whole cell amount (dapi-stained nuclei) was calculated with Fiji/tresholding by detecting high CK5 signal within the cytoplasm. Graph shows the amount of highly CK5 positive cells (%) in ctrl and TRPV4 KO cell lines. (**I)** 184A1 Mammary epithelial cells were treated with either ctrl siRNAs or TRPV4 targeting siRNA on plastic dishes. Cellular lysates were performed after 4 days of siRNA treatments and CK5 was detected with Western Blotting. GAPDH was used as a loading control. (**J)** Quantification of the Western Blots, related to (**I**). n(CK5, ctrl) = 3; n(CK5, siRNA) = 3.

TRPV4 has been shown to play a role in the differentiation of fibroblasts, distinct epithelial and immune cell populations as well as in the differentiation of bone cells^37,62–65^. Our findings indicated that TRPV4 is expressed at high levels in the stem and progenitor cell populations of the mammary epithelial cells, suggesting that it may also play a role in the lineage specific differentiation in these cell types. As expression of TRPV4 is dependent on the mechanical features of the cell microenvironment and there are indications that TRPV4 activity is also regulated through biophysical changes (Fig. 1G,H^65,66^), we performed experiments on various compliances to investigate the possible role of TRPV4 in stiffness-dependent differentiation of the mammary epithelial cells. For this, we used 184A1 breast epithelial cell line for TRPV4 depletion by specific siRNAs or alternatively depleting TRPV4 by siRNA or CRISPR from MCF10A cells (Fig. S2). Both MCF10A and 184A1 cell lines contain stem/progenitor cells as well as mature ME and LE cells^67^. Culturing 184A1 cell line on different stiffnesses, revealed a rigidity-dependent change in the proportion of LE and ME cell types (Fig. S3A–C). This finding was based on the expression levels of specific markers for myoepithelial cells (α-SMA, Vimentin, P-cadherin), luminal epithelial cells (CK18 and E-cadherin) and for stem/progenitor cells (Slug). However, depletion of TRPV4 caused alterations in the levels and stiffness-dependent differentiation pattern of the specific cell types (Fig, S3A–C, right panel). Depletion of TRPV4 in the compliant 2D matrices did not have so much impact on the expression levels of myoepithelial markers Vimentin and P-cadherin (Fig. 2A,B). However, a significant decrease in their levels was detected on stiffer environment, i.e. on plastic cell culture dishes (Fig. 2A,B). In contrast, CK18 levels were not significantly altered, while E-cadherin levels were altered both on soft and rigid environment upon TRPV4-depletion (Fig. 2A,B). In addition, immunofluorescence stainings of TRPV4 CRISPR KO cells with p63, a nuclear marker for the mature myoepithelial cells, showed lower amount of p63 positive nuclei as well as lower mean intensity level of p63 in TRPV4 KO cells (Fig. 2C,D), suggesting that TRPV4 plays a role in the differentiation of the mammary epithelial cells.

As Slug protein has been thought to act as the main determinator of the lineage specific differentiation of the mammary epithelial cell populations, we verified the level of Slug in both 184A1 and MCF10A cells, depleted for TRPV4 by siRNAs or CRISPR, respectively. Short term depletion of TRPV4 by siRNAs on either soft or stiff environment did not alter Slug levels (Fig. 2E,F), while long term KO led to slightly but not significantly lower levels (Fig. S4A). We also verified the levels of CK5, a marker for the stem and progenitor cells, directing differentiation of the mammary epithelial cell populations^67^. Interestingly, the amount of both CK5 positive cells and CK5 levels were decreased upon TRPV4 depletion (Fig. 2G–J), implicating that mechanosensitive TRPV4 channel could guide the stiffness-dependent differentiation of the mammary epithelial cell populations through CK5.

### TRPV4 associates with mammary epithelial junctions and maintains epithelial integrity

**Figure 3:**
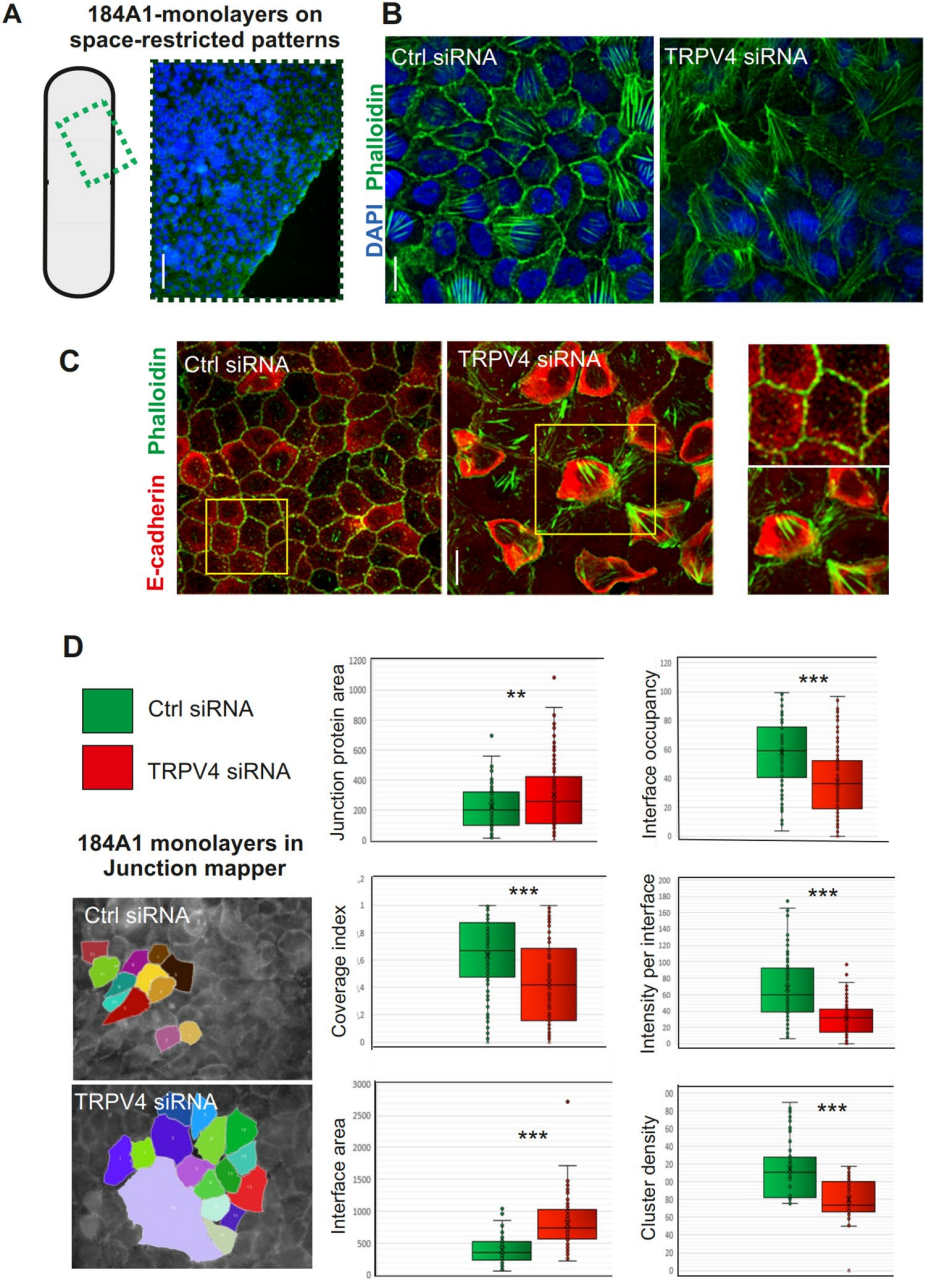
TRPV4 depletion leads to abnormal morphology and junctional disruption of the epithelial sheets. (**A**) 184A1 mammary epithelial cells were cultured on a space-restricted 2D-area to reach rapid confluency of the cultures. Immunofluorescence stainings were performed to detect morphology of the cells within the cultures. Phalloidin visualizes actin cytoskeleton (green) and DAPI nuclei (blue). Bar 140 um. (**B)** Magnifications of the stainings, related to (**A**). Phalloidin-green; DAPI-blue. Bar 20 um. (**C)** Immunofluorescence stainings of 184A1 cell cultures on space-restricted patterns. Specific antibody was utilized to visualize E-cadherin (red) at the cell–cell junctions. Phalloidin was used for visualizing actin cytoskeleton (green). Magnifications of the indicated regions (yellow boxes) are shown on the right side of the panel. Bar 20 um. (**D)** Junction Mapper analyses^68^ was used to screen differences in the parameters related to junctional integrity in ctrl and TRPV4-depleted 184A1 cell cultures. Examples on representative epithelial sheets, analysed with the software. Colored regions indicate analysed cells. On the right side panel, differences in junction protein area, interface occupancy and area of the junction protein, coverage index, intensity per interface, and cluster density in between ctrl and TRPV4 siRNA-treated 184A1 epithelial sheets are shown with box blots with inner and outer points and mean. n(ctrl) = 112, n(TRPV4 siRNA) = 142. **P < 0.01; ***P < 0.001 (t-test two-tailed, two sample equal variance).

Loss of TRPV4 played a role in the lineage specific differentiation of the mammary epithelial cells and additionally led to decreased levels of E-cadherin. To assess how these changes could be reflected to the structural features of the epithelial sheets, we did further studies on the role of TRPV4 in mammary epithelial monolayers. For this, either 184A1 or MCF10A cells were depleted for TRPV4 (Fig. S2). Such cells were cultured on 2D-space restricted patterns to reach rapid confluency of the epithelial sheets (Fig. 3A). Early after attachment and spreading, we noticed a delay in the spreading of the single epithelial cells (Fig. S4B). Additionally, such cells displayed altered morphological features, as can be seen from the decreased anisotropy and increased surface area at the monolayer edges in comparison to the control cells (Fig. S4C,D). These epithelial sheet cultures on space-restricted patterns were further stained with phalloidin and E-cadherin for analyses with junction mapper program^68^ to monitor several parameters that are linked to epithelial integrity through features of the cell junction area. Based on these analyses, TRPV4 has a clear role in the maintenance of intact epithelial cell–cell junctions (Figs. 3B–D, S5A,B). This was also evident from the E-cadherin stainings, which were showing a less prominent junctional pattern with more diffuse protein or nascent adhesion like protrusive cell–cell junctions (Figs. 3C, S5C).

### TRPV4 depleted mammary epithelial cells lose their contractile potential and normal 3D structures

**Figure 4:**
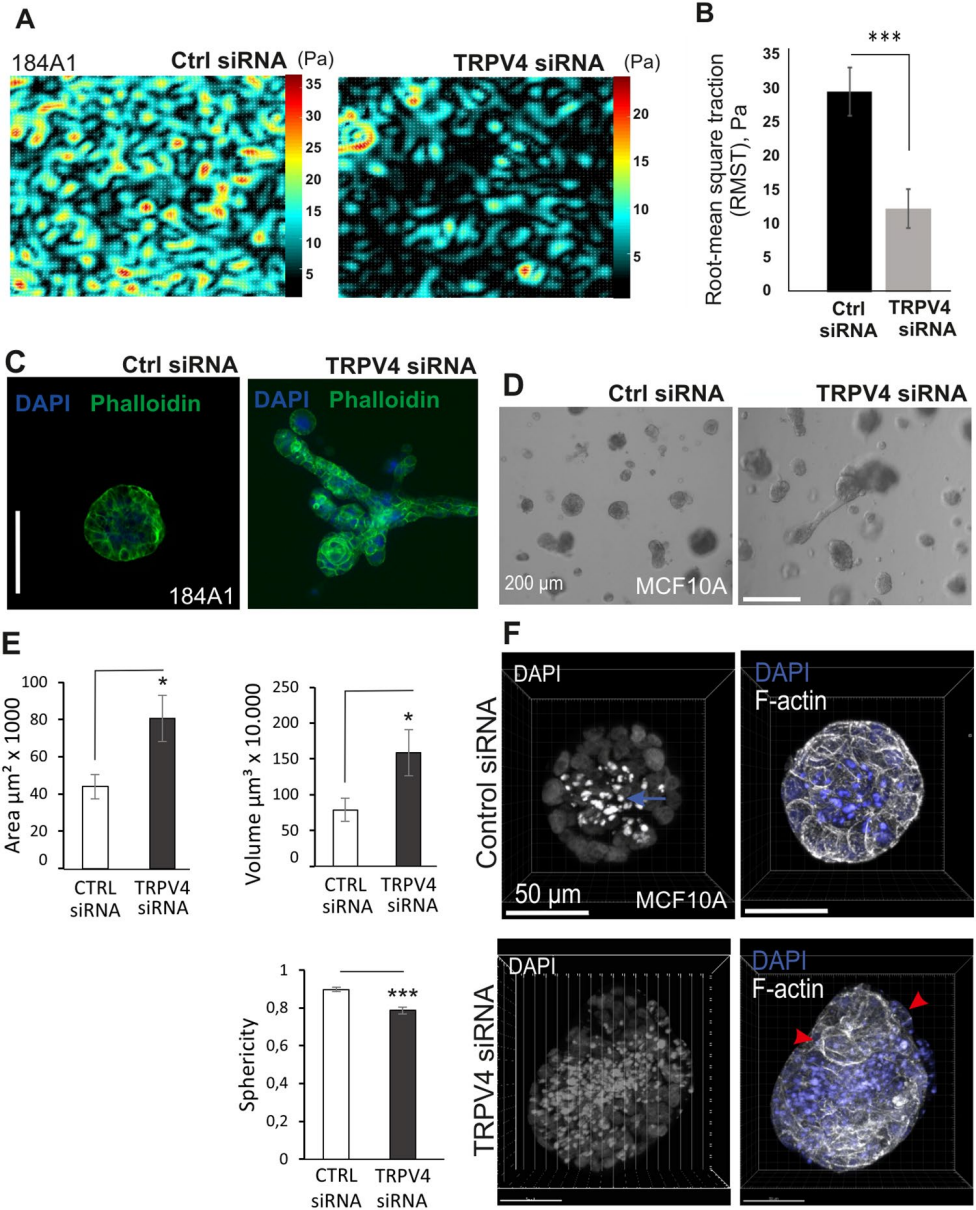
TRPV4 guides epithelial actomyosin forces and its depletion leads to impaired maintenance of 3D mammosphere structures. **(A)** 184A1 mammary epithelial cells were cultured to confluency on 96-well plates designed for traction force imaging. Monolayer forces were monitored for both ctrl epithelial sheets and for epithelial sheets depleted for Trpv4 with specific siRNA. Examples of representative force maps are shown in the image. (**B)** Mean of root-mean square tractions (RMST) in Pascals (Pa), related to (**A**) are shown. n(ctrl siRNA) = 21, n(TRPV4 siRNA) = 22; Mean (+ /− SEM) is shown. ***P < 0.001 (t-test, two-sample equal variance). (**C)** Immunofluorescence, IF, stainings of 184A1 mammary epithelial cells in 3D Matrigel after three weeks of incubation. Ctrl cells were treated with scrambled siRNA and TRPV4 was depleted with specific siRNA against TRPV4 every fourth days. Phalloidin is visualized with green color and DAPI (nuclei) with blue. Bar 100 um. (**D)** MCF10A in 3D Matrigel cultures after 3 weeks of incubation with either ctrl or TRPV4 siRNAs. Brightfield images are shown, bar 200 um. (**E)** Quantifications of the area, volume and sphericity from the MCF10A 3D cultures, related to (**D**). Data represent mean + /− SEM, n(ctrl siRNA) = 20, n(TRPV4 siRNA) = 20; *P* < 0.05 *; *P* < 0.001 *** (unpaired two-sample Student’s t-test). (**F)** 3D reconstituted confocal immunofluorescence (IF) images of ctrl siRNA and TRPV4 siRNA-treated MCF10A cells in Matrigel. Nuclei were stained with DAPI (blue) and filamentous actin (F-actin) stained with Phalloidin (white). In spheroid samples nuclear fragments (blue arrow) are located evenly in the center, while in TRPV4-depleted sample nuclear fragmentation is more random and spheres vary morphologically, with some cellular protrusions (red arrowheads). Bar 50 um.

Cellular forces are crucial for the maintenance of epithelial cell–cell junctions and in this way epithelial integrity^69–71^. To investigate whether the disrupted cellular junctions were associated with alterations in the cellular forces, we performed traction force imaging on epithelial sheets. Monolayer force microscopy was performed on 96-well dishes, covered with compliant 1 kPa silicone matrix with embedded nanobeads. Comparing the cellular forces, applied by either control or TRPV4-depleted cellular sheets, showed significantly lower forces upon TRPV4 depletion (Figs. 4A,B, S6A,B). This was evident in both TRPV4 siRNA and TRPV4 CRISPR cell lines. Furthermore, analyzing the proteins related to actomyosin assembly (p-Thr18/Ser19-MLC, p-Thr172-AMPK and P-Thr286-CaMKII), we could detect alterations in the activity of these proteins, suggesting that TRPV4 could be upstream of the pathways linked to cellular actomyosin contraction (Fig. S6C–F).

**Figure 5:**
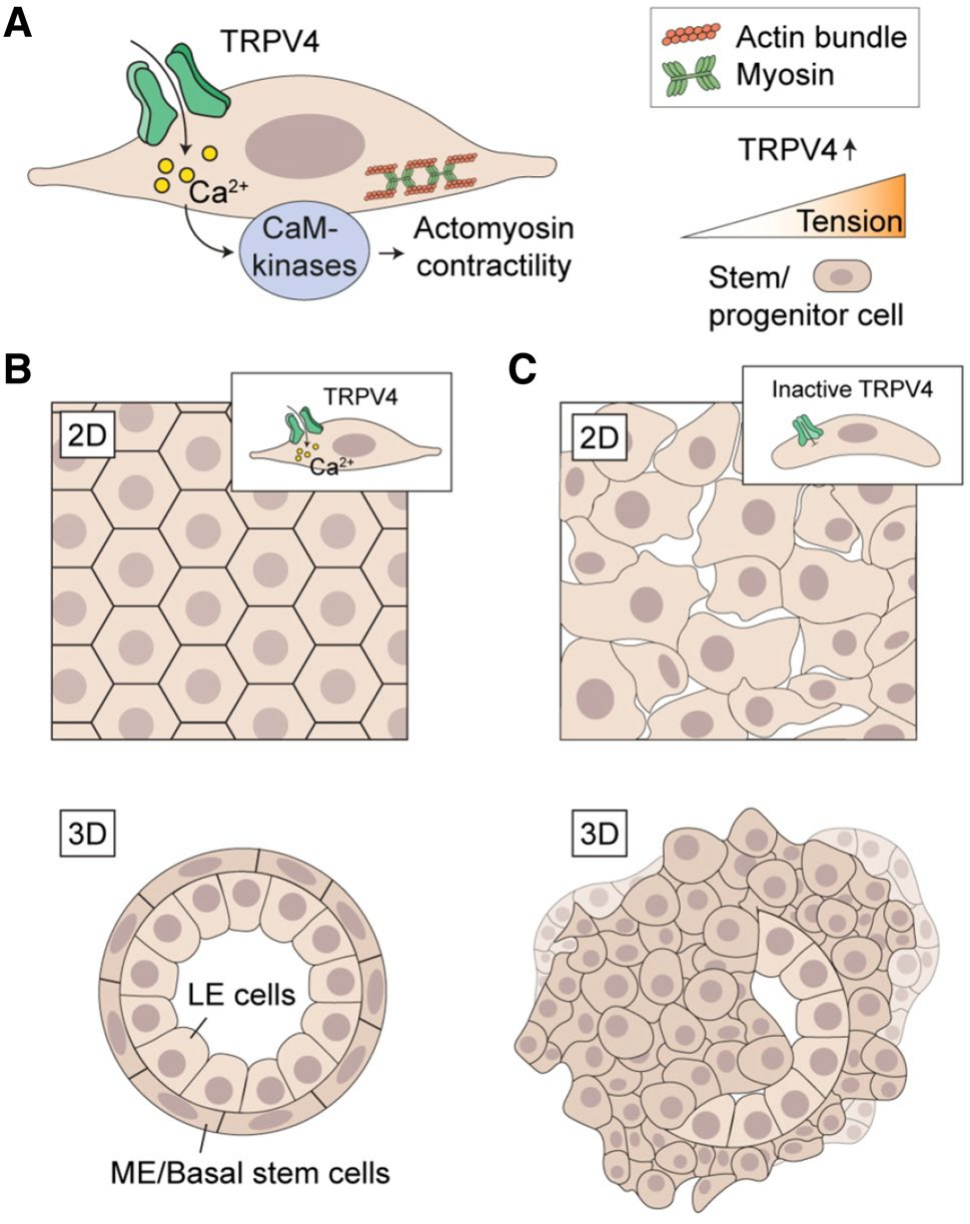
Hypothetical function of TRPV4-channel in the maintenance of epithelial integrity. **(A)** Ion influx through mechanosensitive TRPV4 channel affects various cellular processes through its impact on intracellular cascades. Ca^2+^-influx impacts for instance mechanical features of the cells by playing a role in cellular contractility through several kinases upstream of actomyosin assembly. Such kinases are for example Ca^2+^-dependent CaM Kinases. (**B)** Actomyosin contractility itself has a major role in the maintenance of intact epithelial junctions through regulation of tension at the cell–cell contacts. This is important f.i. for the maintenance of junctional E-cadherin. Normal TRPV4 function thus appears to be important for mammary epithelial structures both in 2D and 3D. (**C)** Loss of TRPV4 from the mammary epithelial cell populations subsequently leads to disrupted mechanical features and impaired 2D and 3D mammary epithelial structures. Normally, mammary epithelium is bilayered and formed by inner luminal epithelial- and outer, contractile myoepithelial cell layer (B, lower picture). The bipotent mammary stem cells locate in the outer myoepithelial cell layer and are exposed to various biophysical changes of the microenvironment. TRPV4 ion channel is one of the mechanosensors, prominently expressed in the basal stem cells and impacting cellular processes via physical changes in the microenvironment. Tension itself is also directly regulating TRPV4 expression. TRPV4 could thus be a major regulator of the differentiation of distinct mammary epithelial cell populations via extracellular mechanical changes. It may thus have a dual function in the regulation of mammary epithelial structures: Through its role in the regulation of actomyosin contractility and epithelial integrity, as well as through its role in cell differentiation.

Mammary myoepithelial cell layer is highly contractile, and its force-producing features must be crucial for the maintenance of normal 3D morphology in the bilayered mammary epithelium. As we saw a change in the cellular force production as well as an impaired differentiation of the mature, contractile myoepithelial cells after depletion of TRPV4, we wanted to investigate further the possible role of TRPV4 in the maintenance of normal 3D structures. To analyze whether the deregulated cellular force-production could also be reflected to the morphology of the corresponding 3D structures, we performed 3D Matrigel cultures with both MCF10A and 184A1 cells, treated with either ctrl siRNA or TRPV4 siRNA (Fig. 4C–F). In both cases, downregulation of TRPV4 led to atypical 3D mammosphere structures with higher volume and area, as well as decreased sphericity (Fig. 4E). Taken together, TRPV4 plays a role in the cellular force production either by directing the differentiation of α-SMA-expressing contractile myoepithelial cells and/or directly affecting pathways above actomyosin contractility. The state of cellular contractility itself is in turn important for the maintenance of normal cellular junctions and 3D morphology. The hypothetical model for the role of TRPV4 in the mammary epithelium is presented in Fig. 5.

## Discussion

Biophysical features of the stroma are recognized by various cell membrane-embedded mechanosensitive machineries that convert physical information into biological responses^1,2^. One of those mechanosensors is TRPV4 calcium channel that is widely expressed in various tissues and has a major role in the regulation of calcium-dependent signaling cascades upon extracellular cues^12,14,15,17–20^. Subsequently, TRPV4 can direct a wide range of physiological functions.

In the mammary gland, TRPV4 levels have been shown to increase significantly at Day 15 of gestation and decline shortly after lactation^10^. Since it is also activated by temperature and mechanical forces that guide development of mammary epithelial structures, TRPV4 has been proposed to play an important role in the formation of normal mammary gland. Here we report, that TRPV4 is highly expressed in the mammary stem-and progenitor cells, based on the co-expression with CK5 and Slug proteins (Figs. 1, S1). Such localization suggested a functional role in these cell populations and in line with this hypothesis, TRPV4 depletion led to defects in the maturation of myoepithelial cells, in particular (Figs. 2, S3 and S4). Accordingly, TRPV4 has been shown to regulate the differentiation of many other cell types: TRPV4 is known to e.g. direct the differentiation of cardiac and airway fibroblasts as well as dermal myofibroblasts^62,65,72,73^. In the case of cardiac fibroblasts, TRPV4-dependent differentiation seems to be orchestrated by both biochemical and mechanical cues and involves actomyosin-regulating Rho kinase pathway^37,62^. Similarly, TRPV4 depletion from the mammary epithelial cells affected pathways upstream of actomyosin assembly (Fig. S6). Additionally, in fibroblast differentiation, TRPV4 has been shown to regulate the levels of α-SMA through transcription coactivator, myocardin-related transcription factor-A, MRTF-A in a stiffness dependent manner^63^. In agreement with this, in mammary epithelial cells TRPV4 affected differentiation of α-SMA-expressing myoepithelial cells stiffness-dependently and the levels of TRPV4 itself were also dependent on the rigidity of the underlying matrix (Figs. 1, 2, S3, S4).

Besides mammary epithelial cells, TRPV4 has been linked to the differentiation of other epithelial cell types. In a mouse model, TRPV4 was shown to impact differentiation of lung epithelial cells^74^. It may also be that in the lungs, the ability of TRPV4 to sense mechanical changes in the cell microenvironment is essential for the morphogenesis of all the tissue compartments within the lungs, including epithelium, smooth muscle layer and the vascular compartment^75^. Furthermore, TRPV4 plays a role in the normal differentiation of corneal epithelium, urothelium and kidney epithelium^30,76,77^. Mechanosensitive features of TRPV4 are additionally needed for the differentiation and activation of immune cells^66,78^. Furthermore, mechanosensory role of TRPV4 and its activation by mechanical forces is known to direct homeostasis of several musculoskeletal tissues: Both the composition and mechanical loading regulate joint chondrogenesis in a TRPV4-dependent manner^79–82^ and mechanical forces contribute to the osteogenic differentiation of human mesenchymal stem cells^64,83–86^. TRPV4-mediated mechanosensitive calcium influx may thus regulate differentiation of several cell types.

How does TRPV4 then direct the differentiation of mammary epithelial cell populations from their bi-potent progenitors? Most likely alterations in the stromal composition and rigidity play a major role in this process as the differentiation of luminal and myoepithelial cells depend on the stiffness of the environment^87^ (Fig. S3). Additionally, TRPV4 levels respond to the tension from the microenvironment and TRPV4-depletion-induced differentiation bias in the mammary epithelial cell populations takes place in a stiffness-dependent manner (Figs. 1, 2). Major changes in myoepithelial markers Vimentin, P-cad and α-SMA were detected primarily on rigid surfaces (Fig. 2). Also, total amount and levels of p63 positive nuclei were decreased upon deprivation of TRPV4 (Fig. 2). p63 is required for the maintenance of mammary epithelial cells in a myoepithelial state and forced p63 expression turns embryonic multipotent progenitors into unipotent mammary basal cell types^88–90^. As in other cell types p63 expression is determined by the stiffness of the ECM through Rho/ROCK-dependent pathway^84,91,92^, the mechanisms behind TRPV4-induced maturation of p63 positive cells could take place in a similar manner in the mammary epithelium. Besides p63, also Slug has been shown to be essential for the lineage-specific determination within the breast epithelium^93^. However, we did not detect any significant alterations in its levels after TRPV4 depletion (Figs. 2, S4). In turn, we detected changes in the levels of CK5, a stem and progenitor cell marker, which in our previous studies was found to act as a regulator of the lineage-specific differentiation of the mammary epithelial cells^67^ (Fig. 2). CK5 itself seems to react to the mechanical status of the cells^67^, possibly through TRPV4-mediated signaling.

Besides affecting lineage-specific differentiation of the mammary epithelial cells, we found that TRPV4 was important in the maintenance of normal cell–cell junctions (Figs. 3, S5). This was evident by the analyses of cellular monolayers, formed by either ctrl or TRPV4-depleted 184A1 or MCF10A cell cultures, which were analyzed after immunofluorescence stainings with the junction mapper program. Several parameters related to the integrity of the epithelium, were disturbed (Figs. 3D, S5A). Additionally, TRPV4 localized with E-cadherin based adherens junctions and the levels of E-cadherin and P-cadherin were significantly decreased upon TRPV4 depletion, leading to more diffuse E-cadherin in the cytoplasm (Figs. 1E, S1C,D; Figs. 2A,B, 3C). This was also reflected to the anisotropy and the area of the cells (Fig. S4C,D). In line with these findings, TRPV4 has been shown to be crucial for the adhesive complexes of some other cellular systems as well: It plays a role in the maintenance of alveolar epithelial barrier function of the lungs^94^ and regulates adherens junctions in the urothelial epithelium and oral epithelium^29,33^. Additionally, TRPV4 KO leads to leaky vessel through the regulation of VE-cadherin^95^. Furthermore, TRPV4 can regulate junctional integrity also through the maintenance of tight junctions as shown in the corneal epithelium and mammary epithelium models^30,32^. As TRPV4 depletion, in the mammary epithelial cells, led to alterations in the pathways upstream of actomyosin assembly and consequent lower cellular force production, this could be one reason for the loss of epithelial integrity (Figs. 4A,B, S6). In addition, TRPV4 has been connected to the activation of specific integrins^96^, which may also impact cellular force production. The loss of epithelial integrity together with the altered ratio of differentiated myoepithelial and luminal epithelial cells, were reflected clearly to the 3D morphology of the TRPV4 KD cultures (Fig. 4C–F). As TRPV4 functions also as a thermosensor and changes in body temperature have been linked to the regulation of epithelial barrier function in the skin keratinocytes through TRPV4-induced expression of E-cadherin and reorganization of the actin cytoskeleton^97^, it would also be relevant to study the link between temperature changes and epithelial barrier function in the mammary epithelial model.

While in this study we have investigated the role of TRPV4 for the maintenance of normal mammary epithelial sheets, most of the previous studies have concentrated on the role of altered TRPV4 expression along cancer progression, including its role for the development of various breast carcinoma types^98^. As progression of breast carcinomas involves increased stiffening of the stroma^99–101^, our findings that TRPV4 is regulated by the mechanical features of the microenvironment and regulates differentiation of mammary epithelial cell populations in a stiffness-dependent manner (Figs. 1, 2) may also be linked to cancer progression. In addition, one should consider the interplay between various mechanosensitive ion channels, including the Piezo family channel proteins. TRPV4 and Piezo1 channels are known to mediate separate yet intersecting mechanosensitive pathways^102^, suggesting that regulation of Piezo1 channel could further enhance the effect of TRPV4 depletion in bilayered mammary epithelium. Also, in endothelial cells, activation of Piezo1 by fluid shear stress initiates a calcium signal, leading to the opening of TRPV4. This subsequent opening of TRPV4 is responsible for the prolonged increase in calcium levels, initiating pathological events. Consequently, the effects of shear stress in the cell layer are initiated by Piezo1 but require TRPV4^103^. Abnormal biophysical signaling may thus favor cancer progression through altered signaling via TRPV4 with other cooperative ion channels, and this should be more thoroughly explored in the future.

## Methods

### Cell culture and treatments of cell cultures

MCF10A or 184A1 mammary epithelial cells were purchased from ATCC (ATCC® CRL-10317™) and maintained in DMEM/F12 supplemented as described by Brugge lab (https://brugge.med.harvard.edu/protocols) and elsewhere. The cells were subcultured twice a week, before reaching 80% confluency. TRPV4 siRNA treatments were performed on around 50–70% confluent cell cultures, one day after plating the cells on 35 mm dishes. For siRNA silencing, 25 nM ON-TARGET plus SMARTpool™ siRNA against TRPV4 (#L-004195-00-0010, Dharmacon, GE Healthcare) was transfected into cells by using RiboJuice transfection reagent (#71115, Novagen). Cells were incubated for 3–4 days for efficient depletion of the target protein, TRPV4. Note that both ctrl and TRPV4-depletions were performed in similar manner.

### Cell culture on different stiffnesses

184A1 mammary epithelial cells were plated very sparse (around 10% confluency) on collagen-coated CytoSoft® cell culture dishes with 0.2, 2, 16 and 64 kPa stiffnesses (Merck/Sigma Adrich). Cells were incubated for 12 days to let them reach confluent monolayers. siRNAs were added on the following day, after plating the cells and every fourth day thereafter. After 12 days in culture, cells were lysed for Western blotting.

### 3D cell culture

3D cultures were performed with both MCF10A and 184A1 mammary epithelial cells. For that, growth factor reduced, GFR, matrigel was thawed overnight (o/n) at + 4 °C (#356230, Lot. 1272006 and 6172006, Corning) and 50 µl of Matrigel was added into each well of a pre-chilled 8-well chamber slide (Lab-Tek Chamber Slide with cover glass slide, Thermo-Fisher). After the polymerization of matrigel (varying in between 15 min and 1 h or o/n at + 37 °C) ~ 5000 cells were plated into each well. 3D cultures were grown in the mammary epithelial cell growth medium with 4% matrigel for 22 days. During the incubation time, cells were transfected with TRPV4 siRNAs, with 3 × higher amount of siRNA in comparison to the 2D cultures. Transfections were performed on the days 4, 8 and 19, after the beginning of the 3D cultures, similarly for both ctrl siRNA and TRPV4 siRNA cultures. Renewal of the cell culture medium was performed every 3–4 days and after 22 days, cultures were fixed with 2% PFA and stained for immunofluorescence microscopy.

### Immunofluorescence stainings

MCF10A or 184A1 cells were fixed with 4% paraformaldehyde, PFA, for 20 min at RT and afterwards washed and stored in PBS. Permeabilization and simultaneous blocking of non-specific binding was performed with 0.1% Triton X-100 in Dulbecco-BSA for 5 min. The following primary antibodies were used in the stainings: anti-TRPV4 (#sc-98592, Santa Cruz Biotechnology and #191580, Abcam); mouse anti-E-cadherin (#14472S, Cell Signaling Technologies, CST); rabbit anti E-cadherin (#3195, CST); mouse anti-CK5 (#ab17130, Abcam); rabbit anti-Slug (#9585, CST); mouse anti p63 (#ab735, Abcam); mouse anti-CK14 (#ab7800, Abcam). Primary antibodies were added to the cells diluted in 1% FCS in 0.3% Triton-X100-PBS: in 1:50 dilution for 1 h at RT. After washing with PBS 3 × 5 min, secondary antibodies were applied and incubated in 1:100–1:200 dilution for 45 min -1 h at RT. Phalloidin was added at the same time with the secondary antibodies in 1:200–1:400 dilution. The following Phalloidins were used: Alexa-568 (#A11031), Alexa-488 (#A11034), Alexa-647 (#A22287), Alexa-488 (#A12379), Alexa-488 (#A12379) (Molecular Probes). After this, DAPI nuclear stain was applied in 1:3000–1:5000 dilution for 2 min at RT. The coverslips were washed in PBS and dipped in Milli-Q water before mounting on the glass slides with Mowiol-DABCO (Millipore and Sigma-Aldrich) or Ibidi mounting medium (Ibidi, Munich, Germany). The slides were stored at + 4 prior to imaging.

### Immunofluorescence stainings of 3D cultures

3D Matrigel cultures were washed with PBS and fixed with 2% PFA for 20 min. Permeabilization was performed with 0.25% Triton X-100 in PBS for 10 min. Cultures were blocked at RT in 0.1% BSA, 0.2% Triton x-100 and 0.05% Tween in PBS + 10% goat serum for 1 h. Primary antibodies were incubated at + 4 °C o/n. After 3 × 10 min wash, secondary antibodies together with phalloidin were incubated for 45 min, followed by 10–15 min incubation with DAPI for visualizing the nuclei. Cells were washed and silicon frames were removed to remove the gel into an imaging glass slide. Mounting was done with Mowiol-Dabco by placing a cover glass on the slide. Glass slide was dried at RT o/n before imaging stacks with Leica TCS SP5. Objective used: HCX PL APO 20x/0.7 Imm Corr (water, glycerol, oil) Lbd.bl.

### Area, volume and sphericity of 3D cultures

Bitplane Imaris software was used to reconstitute and analyze confocal 3D stacks of MCF10A mammospheres. For statistical significance, Student’s t-test was done on measured spheroid volume, area and sphericity.

### Junction mapper analyses

Analyses of cell–cell junction from confluent 184A1 or MCF10A cell monolayers was performed with Junction Mapper-tool as in Brezovkjakova et al., 2019.

### Analyzing cell spreading

Cell spreading was calculated by measuring cell area 24 h after plating the ctrl or TRPV4 siRNA cells. Mean + /− SEM was presented in the data.

### Analyzes of anisotropy in TRPV-depleted monolayers

2D cultures were also grown on laminin coated micropattern (#10-020-00-18, Arena A, Cytoo) with different sized disc patterns. Staining was done as with coverslips. 2D cultures were imaged with LEICA DM6000B using 20x/0.7 HC PL APO CS wd = 0.59, 40x/1.25–0.75 HCX PL APO CS Oil wd = 0.10 and 63x/1.40–0.60 HCX PL APO Lbd.bl. Oil wd = 0.10 objectives. Actin cytoskeleton organization was analyzed with ImageJ FibrilTool plug-in (Boudaoud et al. 2014) from spherical micropattern monolayer cultures. Two outermost cell layers were lined from 63 × IF images comprising ¼ of the whole monolayer circle pattern. The ROI area and anisotropy were computed with the FibrilTool. Two-tailed unpaired Student’s t-test was used to reveal statistical significance.

### Immunohistochemistry

IHC stainings were conducted utilizing automated Ventana BenchMark GT immunostaining device with UltraView universal Dab Detection kit 5269806001 Roche. TRPV4 antibody was diluted in Ventana Antibody diluent 5261899001 and incubated 30 min RT. The use of clinical samples has been approved by the Ethics Committee of Pirkanmaa Hospital District, reference no. R07082, sample cohort I.

### Western blotting

Cells were lysed with Lysis Buffer including 1% Triton X-100 in PBS together with Protease inhibitor Cocktail Set III (#539134, Calbiochem) and Phosphatase Inhibitor Cocktail Set II (#524625, Calbiochem) Millipore, Merck Life Science). 4 × LSB-DTT loading buffer (40% glycerol, 4% SDS, 250 mM Tris–HCl, 3% DTT, bromophenol blue) was added to the lysates and the samples were boiled. Samples were loaded into 4–15% Mini-PROTEAN® TGX™ Precast Gels (#456–1084, BioRad). BioLabs protein ladder was used as size marker (#P77125). Separated proteins were transferred onto Immobilon-P (#IPVH85R, Merck Millipore Ltd.). Non-specific binding was blocked with blocking buffer (5% skimmed milk and 5% BSA) for 1 h at RT. The membranes were incubated with the primary antibodies o/n at + 4 degrees. The following antibodies were utilized in Western Blotting: Rabbit anti-TRPV4 (#sc-98592, Santa Cruz Biotechnology; Rabbit anti-TRPV4 (#191580, Abcam), rabbit anti-Phospho-AMPK-Thr172 (#2335, CST), rabbit anti-Phospho-MLC-2-Thr18/Ser19 (#3674, CST), rabbit mab anti- P-Thr286-CaMKII (D21E4, Rabbit mAb #12716, Cell Signaling), rabbit anti-E-cadherin (#3195, CST), rabbit anti-slug (#9585, CST), rabbit anti-CK18-(ab52948), mouse anti-CK5 (#ab17130, Abcam), mouse anti-CK14 (#ab7800, Abcam), rabbit anti-Vimentin (#5741, CST), rabbit anti-P-cadherin (#2189, CST), mouse anti-SMA (#A5228, Sigma) and rabbit anti-GAPDH (#G9545, Sigma). The membranes were washed with 0.1% Tween-PBS and incubated with secondary HRP- conjugated antibody (Anti-Mouse/Anti Rabbit IgG, CST) for 1 h at room temperature, after which the membranes were washed with 0.1% Tween-PBS. Protein bands were visualized with LAS-3000 Imaging System (Fujifilm Corporation, Tokyo, Japan) after incubation with Luminata Crescendo Western HRP substrate (#WBLURO100, Millipore). The WB membranes were cut to thin sections and blotted with several antibodies.

### Imaging of fixed samples

Widefield imaging of fixed cell specimens was conducted with Zeiss Axio Scope.A1 upright microscope equipped with Zeiss Axiocam 506 color camera, Zeiss HXP 200C Fluorescence Illuminator, 100 W halogen illumination and Specimen holder for 76 × 26 mm sliders. Images were captured using Zeiss LD Plan-Apochromat Corr Ph3 40x/0.95, WD 0.25 mm (Air) and Zeiss Plan-Neofluar 63x/1.25, WD 0.10 mm (Oil) objectives, and Zen Blue 3.0 software.

Confocal imaging of fixed samples was conducted with Zeiss LSM 780, Zeiss Cell Observer.Z1 inverted microscope equipped with stage-top incubator with heating and CO2 control, motorized stage for multipoint imaging, fluorescence correlation spectroscopy (FCS) possibility and tunable pulsed laser. Objective used was Zeiss Plan Apo 63x/1.40, WD 0.19 mm (Oil). The Zeiss LSM 780 microscope is equipped with the following detector: 32-channel QUASAR GaAsP PMT array: quantum Efficiency 45%, excitation range 355–660 nm. Images were captured with Zen Black 2012 software.

### Traction force microscopy, TFM

TFM was done with silicone-based Nusil 96 well plates with embedded microspheres. Plates were received from Prof. R. Krishnan (Harvard University, Boston, USA) and were utilized for monolayer force microscopy. Before plating the cells, wells were incubated with cell growth medium for 1–3 h. Plated cells were incubated for 3 h at + 37 °C for attachment. BF images were taken from each cell monolayer and TRITC channels were used to image fluorescent beads on the substrate underneath the cells. After the first set of images cells were trypsinized and a second set of images were taken in a cell-free configuration. These served as reference images. Comparing the first and second set of bead images, we could calculate bead displacement. With the knowledge of bead displacement field, stiffness of the matrix and manual drawing of the cell boundary, we could compute the cell-exerted tractions by utilizing Fourier transform traction microscopy^104^. Imaging was done with Zeiss LSM 780, Zeiss Cell Observer.Z1 inverted microscope and Zeiss Plan Apo 10x/0.45, WD 2 mm (Air) objective. Images were captured with Zen Black 2012 software.

### Supplementary Information

Supplementary Information 1.

Supplementary Information 2.

## Data Availability

This study did not generate any unique datasets or code. All raw data is available on request from the corresponding author.
