# Supplementary material for: Mechanosensitive TRPV4 channel guides maturation and organization of the bilayered mammary epithelium
Source: Sci Rep. 2024 Mar 21;14:6774. doi: 10.1038/s41598-024-57346-x (PMC10957991; doi:10.1038/s41598-024-57346-x)
Supplement: Supplementary file 1 — Supplementary Information 1. [file 41598_2024_57346_MOESM1_ESM.docx]

**SUPPLEMENTARY FIGURES**

**Kärki et al.,** **Mechanosensitive TRPV4 channel guides maturation and organization of the bilayered mammary epithelium**


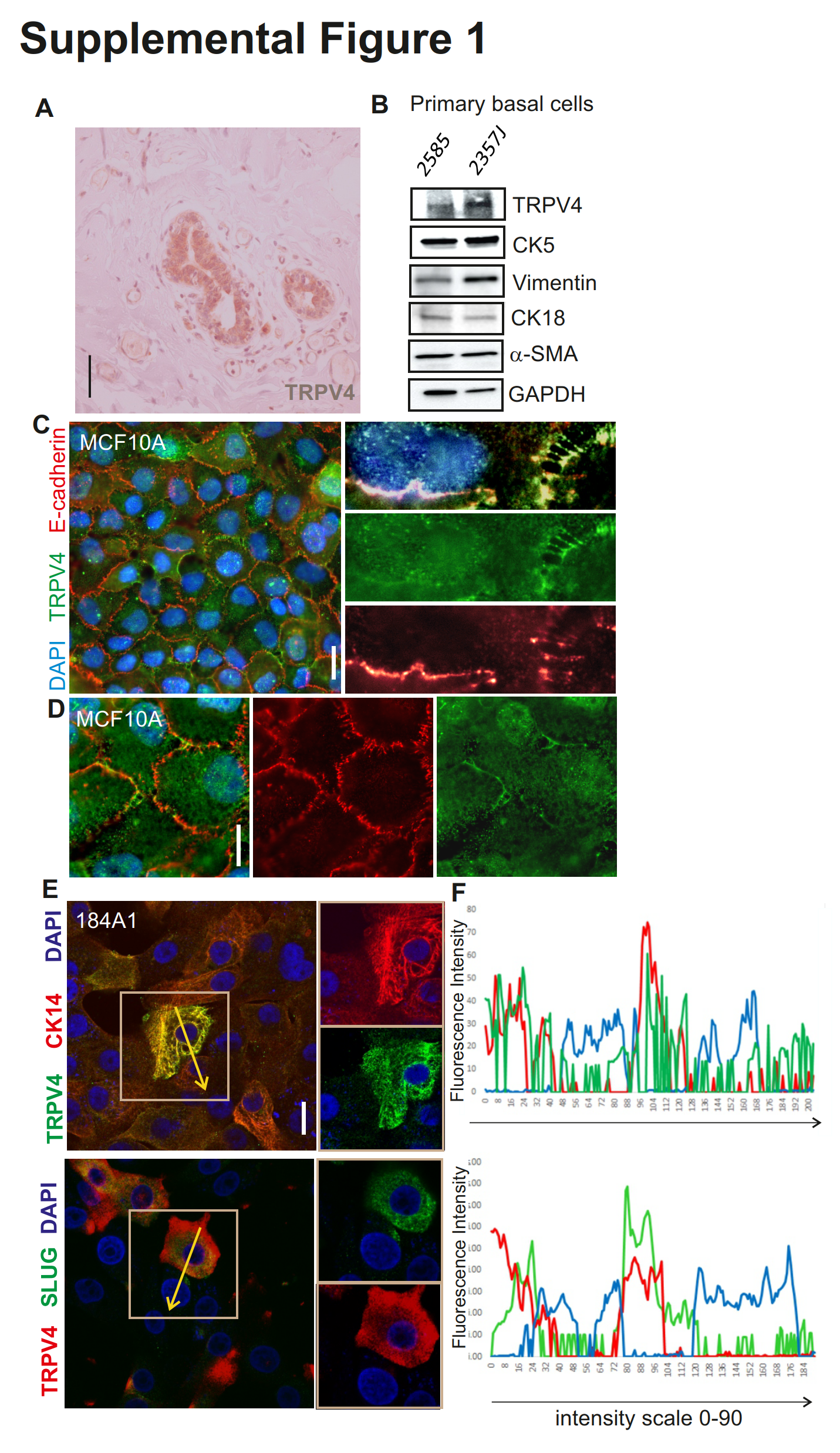


**Figure S1. A)** IHC stainings of a mammary tissue slice with TRPV4 antibody. Bar 200 um. **B)** Detection of TRPV4 levels by Western Blotting (WB) from cellular lysates of FACS-isolated primary basal cells (indicated with running numbers 2585 and 2357J; Breast cancer now tissue bank, London). CK5 indicates the presence of stem and progenitor cells, while Vimentin and α-SMA indicate mature myoepithelial cells and CK18 luminal epithelial cells or their progenitors. GAPDH acts as a loading control. The WB membranes were cut to thin sections and blotted with several antibodies. **C)** Immunofluorescence stainings of semi-confluent MCF10A cell cultures with TRPV4 (green) and E-cadherin (red). Nuclei are visualized with DAPI (blue). Magnification of the indicated region (yellow box) is on the rightside panel. Bar 20 um. **D)** Immunofluorescence stainings of semi-confluent MCF10A cell cultures with TRPV4 (green) and E-cadherin (red). Nuclei are visualized with DAPI (blue). Bar 20 um. **E)** Immunofluorescnece stainings of 184A1 cells with TRPV4 antibody together with progenitor marker CK14 or stem/progenitor marker SLUG, revealed high expression of TRPV4 in the stem-and progenitor cell populations. TRPV4 in the upper panel is indicated with green color and CK14 with red. TRPV4 in the lower panel is indicated with red and SLUG with green. DAPI (blue) indicates nuclei. Bar 20 um. **F)** Lineprofiles of the indicated regions (yellow arrows within the marked boxes in Fig. S1E) showing representative expression patterns of TRPV4 together with either CK14 or SLUG. Distance (x) in pixels.


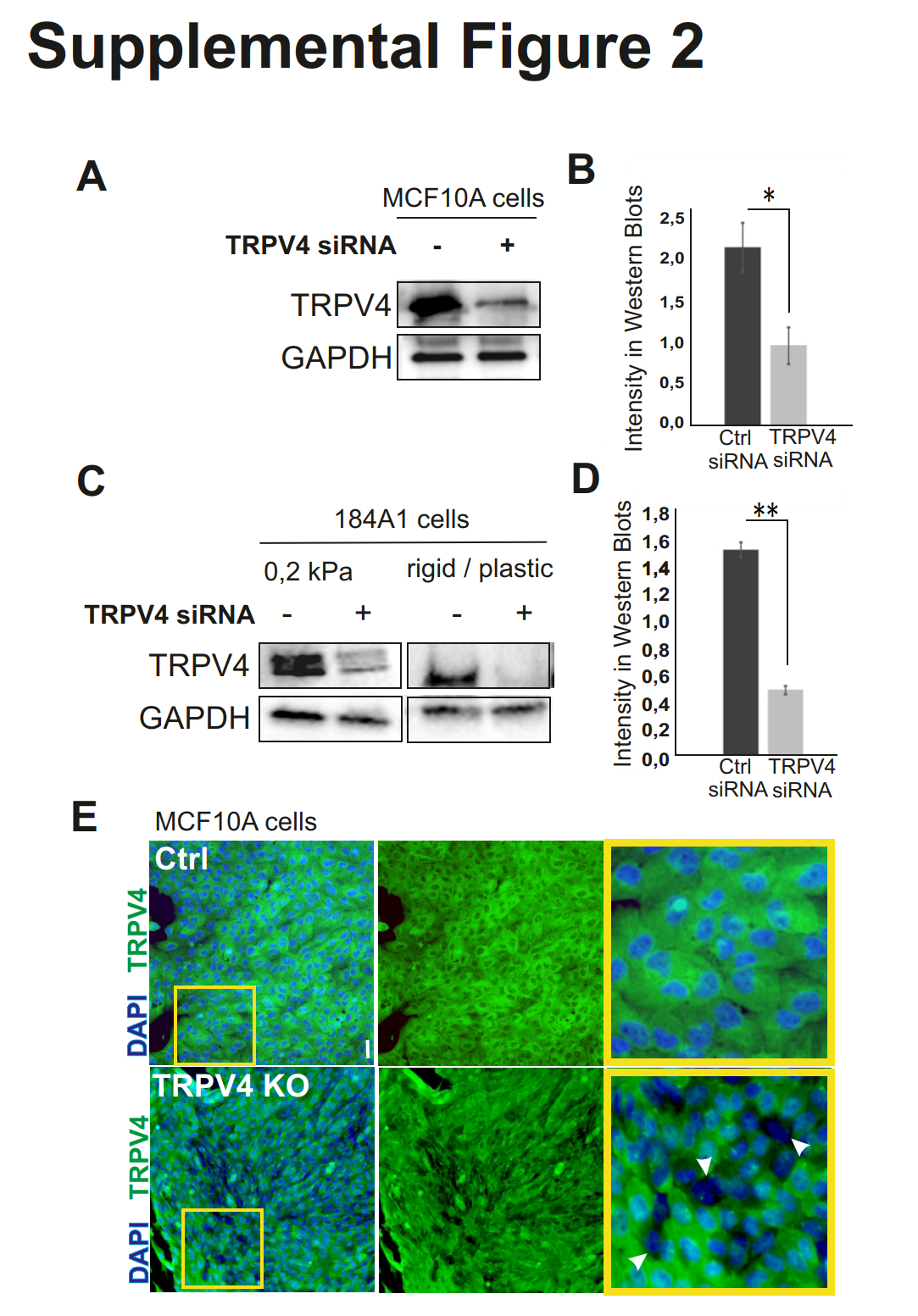


**Figure S2. Depletion of TRPV4 protein. A)** Western Blotting with cellular lysates from MCF10A cells, treated with either control siRNA or specific siRNA against TRPV4. GAPDH acts as a loading control. The WB membranes were cut to thin sections and blotted with several antibodies. **B)** Quantification of the Western Blots, related to Fig. S2A. n=5, mean +/-SEM is shown. *P<0.05 (paired t-test). **C)** Western Blotting with cellular lysates from 184A1 cells, treated with either control siRNA or specific siRNA against TRPV4 and cultures on soft or rigid surfaces. GAPDH acts as a loading control. The WB membranes were cut to thin sections and blotted with several antibodies. **D)** Quantification of the Western Blots, related to Fig. S2C, right panel. n=3, mean +/-SEM is shown. **P<0.01 (paired t-test). **E)** MCF10A cells, depleted for TRPV4 by CRISPR, showed around 15-20% of TRPV KO cells, as demonstrated with the immunofluorescence stainings with specific TRPV4 antibody (green). DAPI was used to visualize nuclei (blue). Examples on KO cells indicated with white arrows. Bar 60 um.


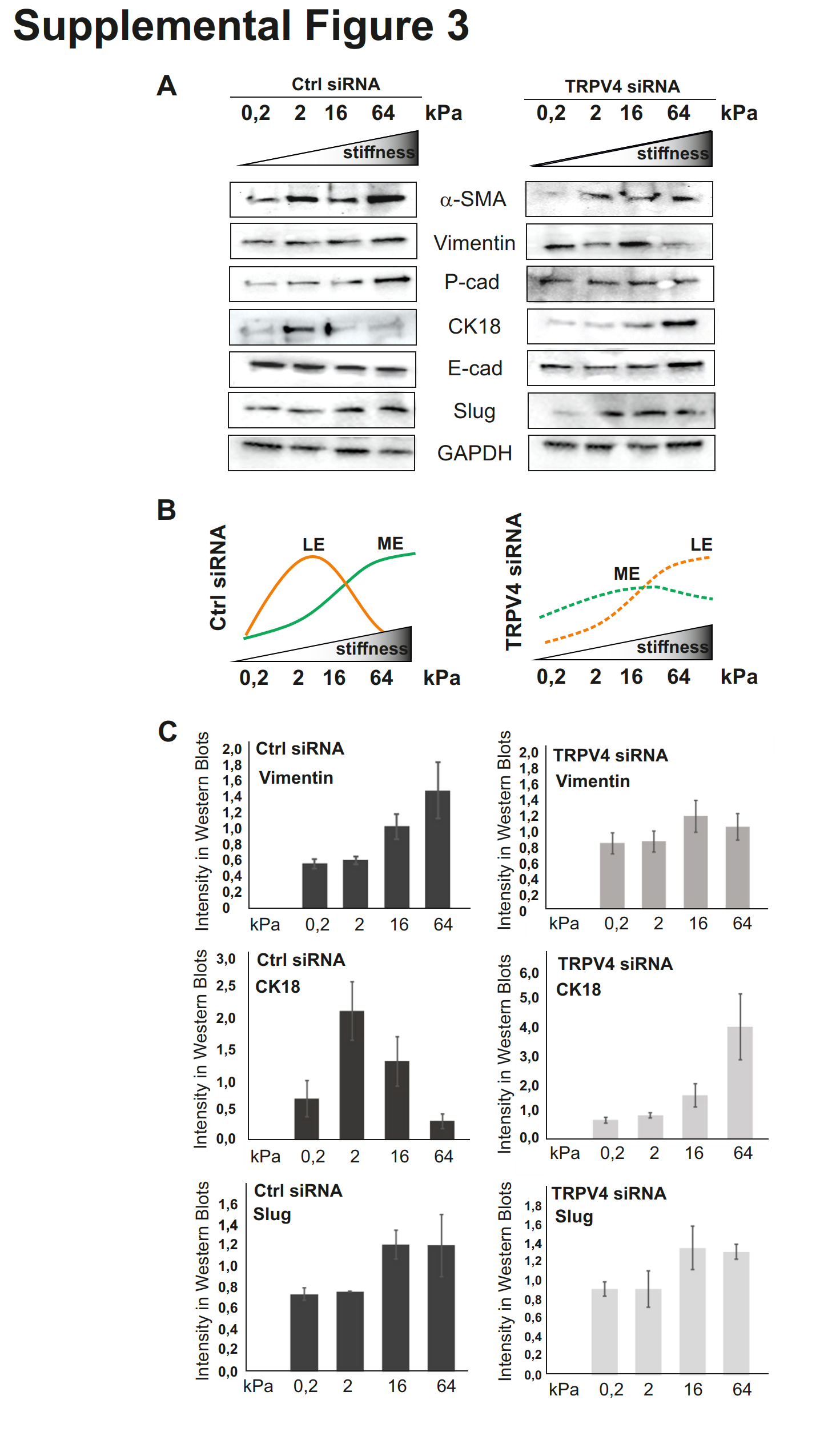


**Figure S3. Differentiation of the mammary epithelial cell populations upon increasing stiffness. A)** 184A1 mammary epithelial cells were treated with ctrl or TRPV4 siRNAs, plated sparse and cultured for two weeks to dense cultures on compliant matrices with 0.2, 2, 16 and 64 kPa stiffnesses. Cells were lysed and protein levels of specific markers were assessed with Western Blotting. The WB membranes were cut to thin sections and blotted with several antibodies. **B)** Illustration of the impact of TRPV4 depletion on the levels of mature myoepithelial (ME) and luminal epithelial (LE) cells. The graphs are based on the typical expression pattern of ME-and LE-specific markers on distinct stiffnesses. **C)** Western blotting from cellular lysates of ctrl siRNA- and TRPV4 siRNA-treated 184A1 cells were performed and levels of vimentin and CK18 were quantified from the marked stiffnesses. n(vimentin, ctrl, 0.2kPa)= 6; n(vimentin, ctrl, 2 kPa)=3; n(vimentin, ctrl, 16 kPa)= 3; n(vimentin, ctrl, 64 kPa)= 7; n(vimentin, ctrl, 0.2 kPa)= 7; n(vimentin, TRPV4 siRNA, 2 kPa)= 3; n(vimentin, TRPV4 siRNA, 16 kPa) = 3; n(vimentin, TRPV4 siRNA, 64 kPa)=7; n(CK18, ctrl, 0.2 kPa)= 3; n(CK18, ctrl, 2 kPa)= 3; n(CK18, ctrl, 16 kPa)= 3; n(CK18, ctrl, 64 kPa)= 3; n(CK18, TRPV4 siRNA, 0.2 kPa)= 3; n(CK18, TRPV4 siRNA, 2 kPa)= 3; n(CK18, TRPV4 siRNA, 16 kPa)= 3; n(CK18, TRPV4 siRNA, 64 kPa)= 3. Mean (+/- SEM) is shown.


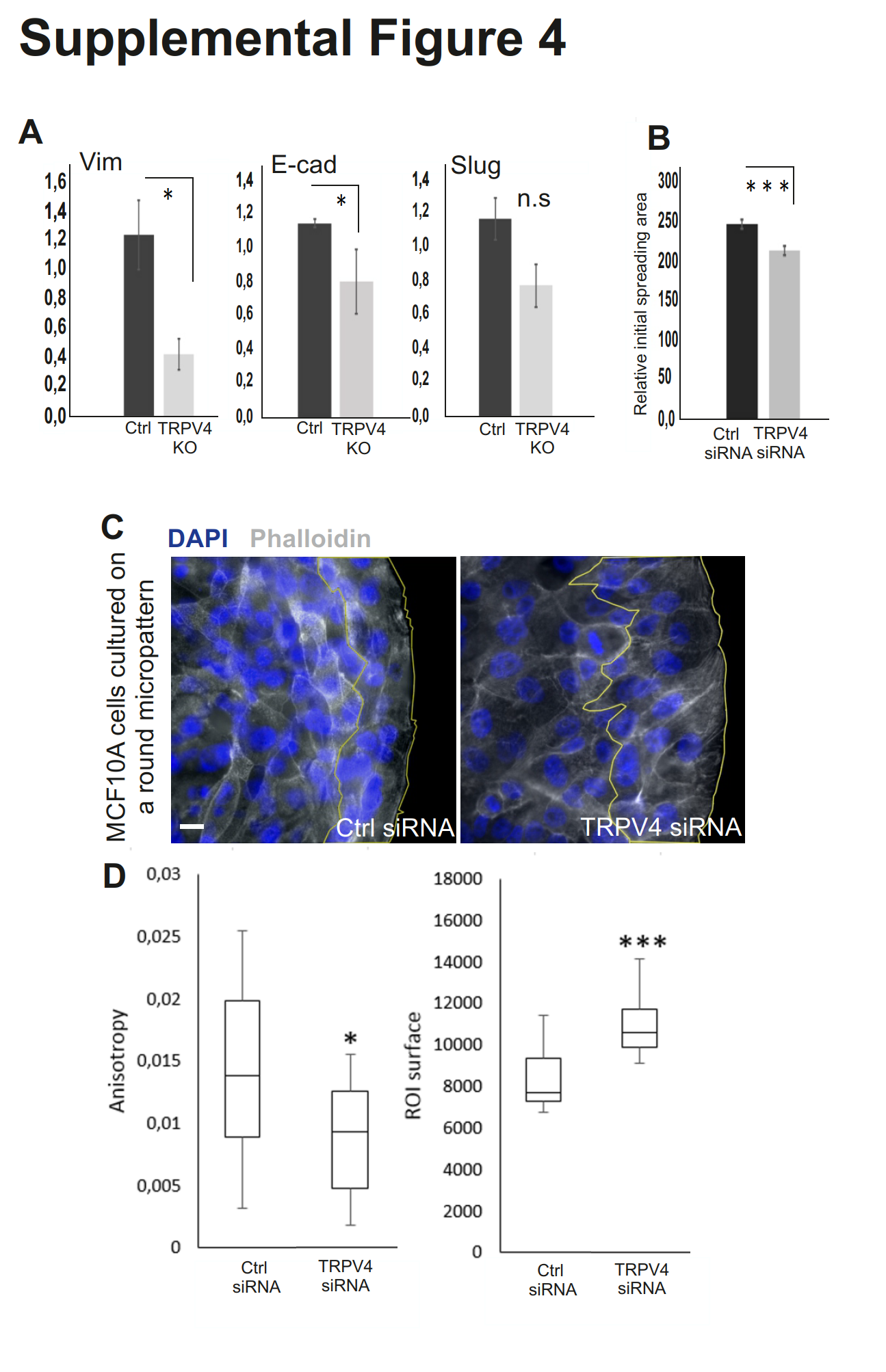


**Figure S4. Alterations in the expression of mammary epithelial markers and cellular morphology upon TRPV4 depletion. A)** n(Vimentin)=4; n(Ecad)= 4; n(Slug)= 4; Mean (+/- SEM) is shown. *P<0.05; n.s.= not significant. **B)** Initial spreading assay in ctrl and TRPV4 depletions; Initial cell spreading assays in ctrl siRNA and TRPV4 siRNA 184A1 cells, cells monitored for spreading 24 h after plating. 2(ctrl)= 525, n(siRNA)= 533; +/-SEM, 3 stars **C)** Immunofluorescence stainings of ctrl and TRPV4 siRNA MCF10A cells, where phalloidin and DAPI were used to visualize cytoskeleton (gray) and nuclei (blue). Two outer layers (yellow borders) of monolayers were analyzed for their fiber anisotropy and area. **D)** Anisotropy and surface area corresponding to S4C. Control siRNA, n = 19, TRPV4 KD n = 24. P < 0.05 *; P < 0.001 ***, unpaired two sample Student’s t-test.


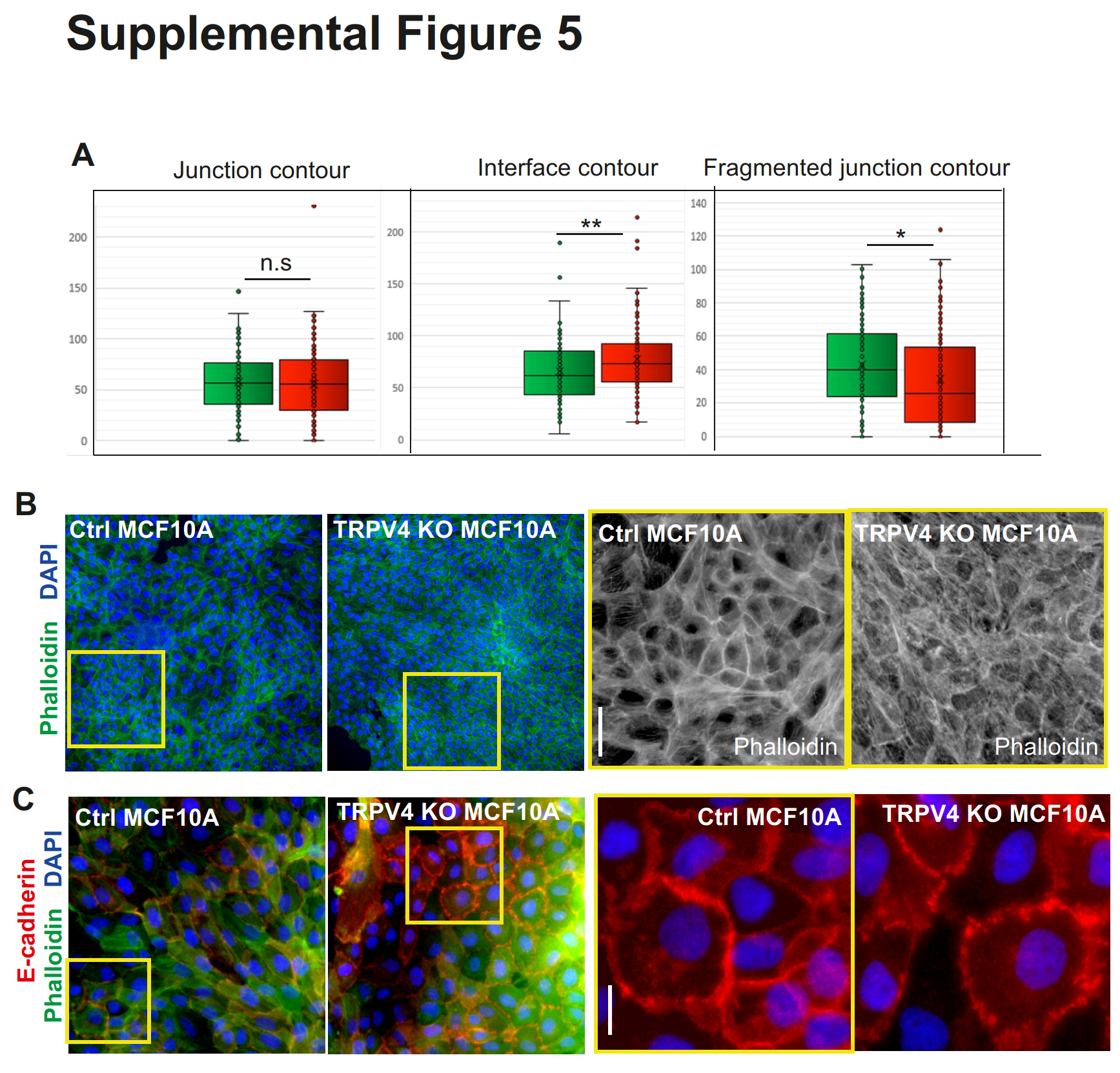


**Figure S5. TRPV4 depletion leads to junctional disruption. A)** Junction Mapper analyses^68^ was used to screen differences in the parameters related to junctional integrity in ctrl and TRPV4-depleted 184A1 cell cultures. Differences in junction and interface contour as well as fragmented junction contour between ctrl and TRPV4-depleted 184A1 epithelial sheets are shown with box blots with inner and outer points and mean. n(ctrl)=112, n(TRPV4 siRNA)=142. *P<0.05; **P<0.01; n.s= not significant (t-test two-tailed, two sample equal variance). **B)** Immunofluorescence stainings of ctrl and TRPV4 KD MCF10A cell cultures. Phalloidin was used to visualize actin cytoskeleton (green) and DAPI nuclei (blue). Magnifications of the indicated regions (yellow boxes) with Phalloidin staining are shown on the right side panel. Bar 60 um. **C)** Immunofluorescence stainings of ctrl and TRPV4 KD MCF10A cell cultures. E-cadherin antibody was used to visualize cell-cell junctions (red), Phalloidin was used to visualize actin cytoskeleton (green) and DAPI nuclei (blue). Magnifications of the indicated regions (yellow boxes) with Phalloidin staining are shown on the right side panel. Bar 20 um.


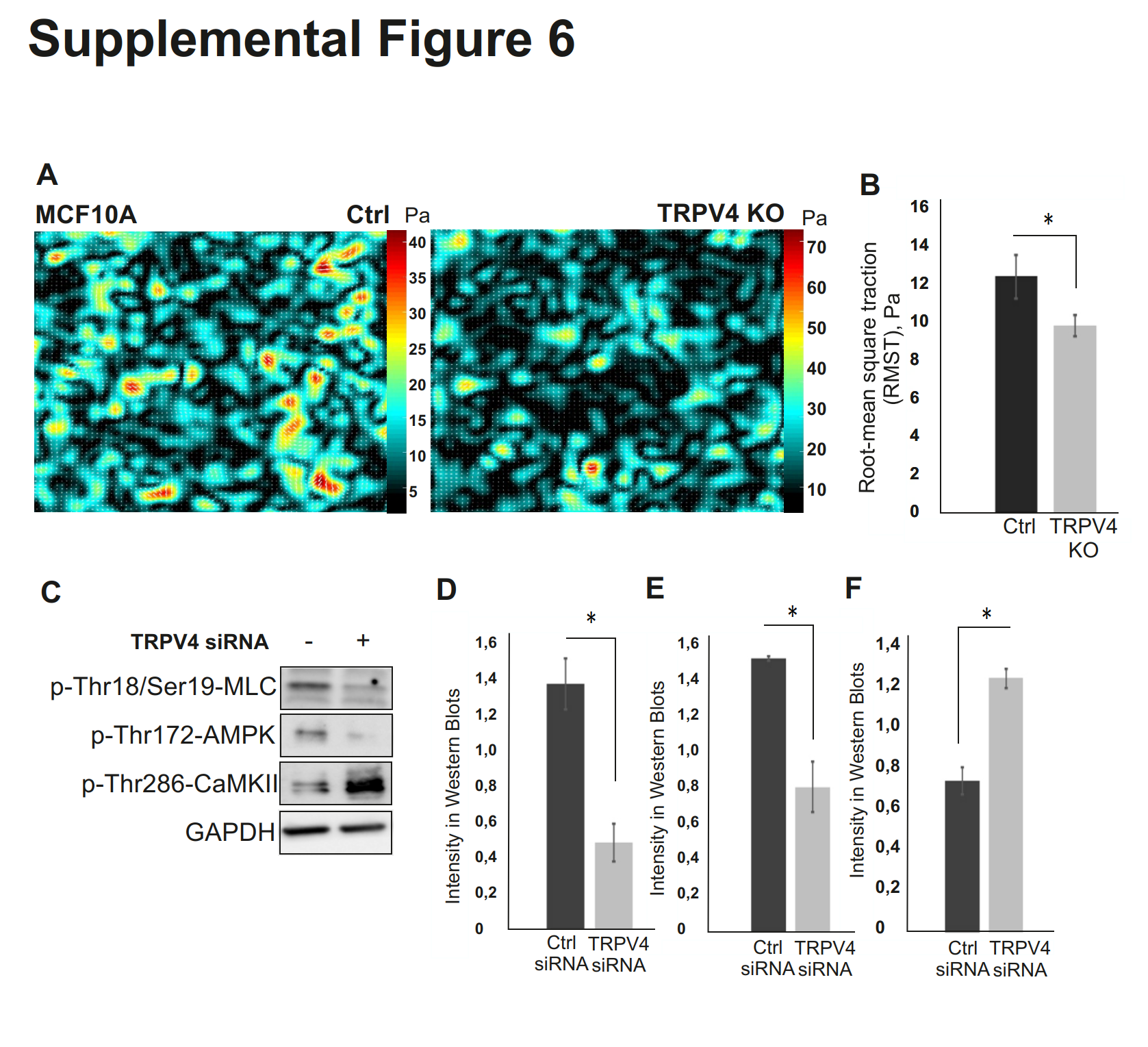


**Figure S6. TRPV4 guides epithelial forces. A)** Ctrl and MCF10A KD mammary epithelial cells were cultured to confluency on 96-well plates designed for traction force imaging. Monolayer forces were monitored for both ctrl epithelial sheets and for epithelial sheets depleted for Trpv4. Examples of representative force maps are shown in the image. **B)** Mean of root-mean square tractions (RMST) in Pascals (Pa), related to Figure 4A are shown. n(ctrl)= 18, n(TRPV4 KD)= 19; Mean (+/- SEM) is shown. *P<0.05 (t-test, two-sample equal variance). **C)** Western Blots of cellular lysates from 184A1 cells, treated with either ctrl siRNA or TRPV4 siRNA, were performed to detect levels of P-Thr18/Ser19-MLC, P-Thr172-AMPK and P-Thr286-CaMKII, related to actomyosin contraction. GAPDH acts as a loading control. The WB membranes were cut to thin sections and blotted with several antibodies. **D)** Quantifications of the Western Blots, related to Figure S6C. Mean (+/- SEM) is shown; n(P-Thr18/Ser19-MLC)=4; n(P-Thr172-AMPK)=4; n(P-Thr286-CaMKII)=4. *P<0.05 (Paired t-test).
