# Supplementary material for: Mechanosensitive TRPV4 channel guides maturation and organization of the bilayered mammary epithelium
Source: Sci Rep. 2024 Mar 21;14:6774. doi: 10.1038/s41598-024-57346-x (PMC10957991; doi:10.1038/s41598-024-57346-x)

Uncropped/unmodified Western Blots

Related to Figure 1F, 1G and S1B

Note that in the experiments we have often cut the WB membranes based on the size marker, so that we can use multiple antibodies for single WB experiments. In many experiments we have also reprobed the membranes with other antibodies if the size of the proteins allows it.

1F

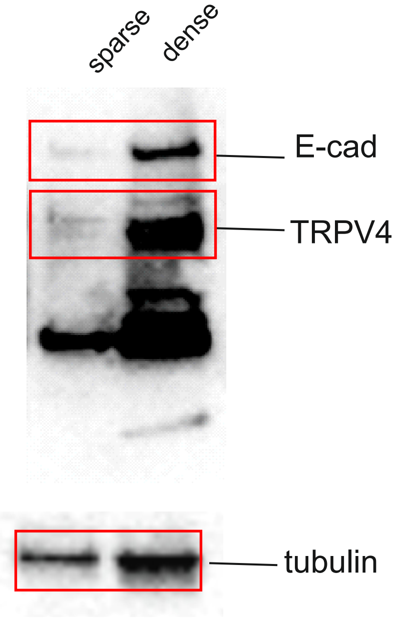

1G

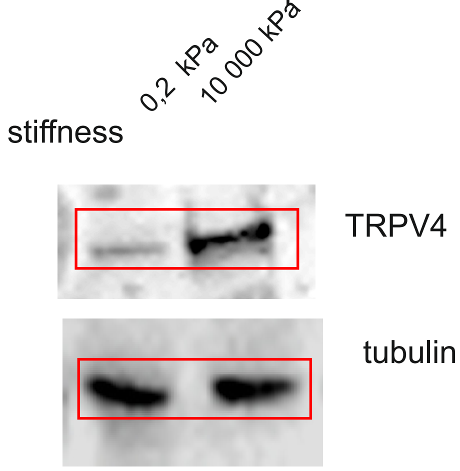

S1B

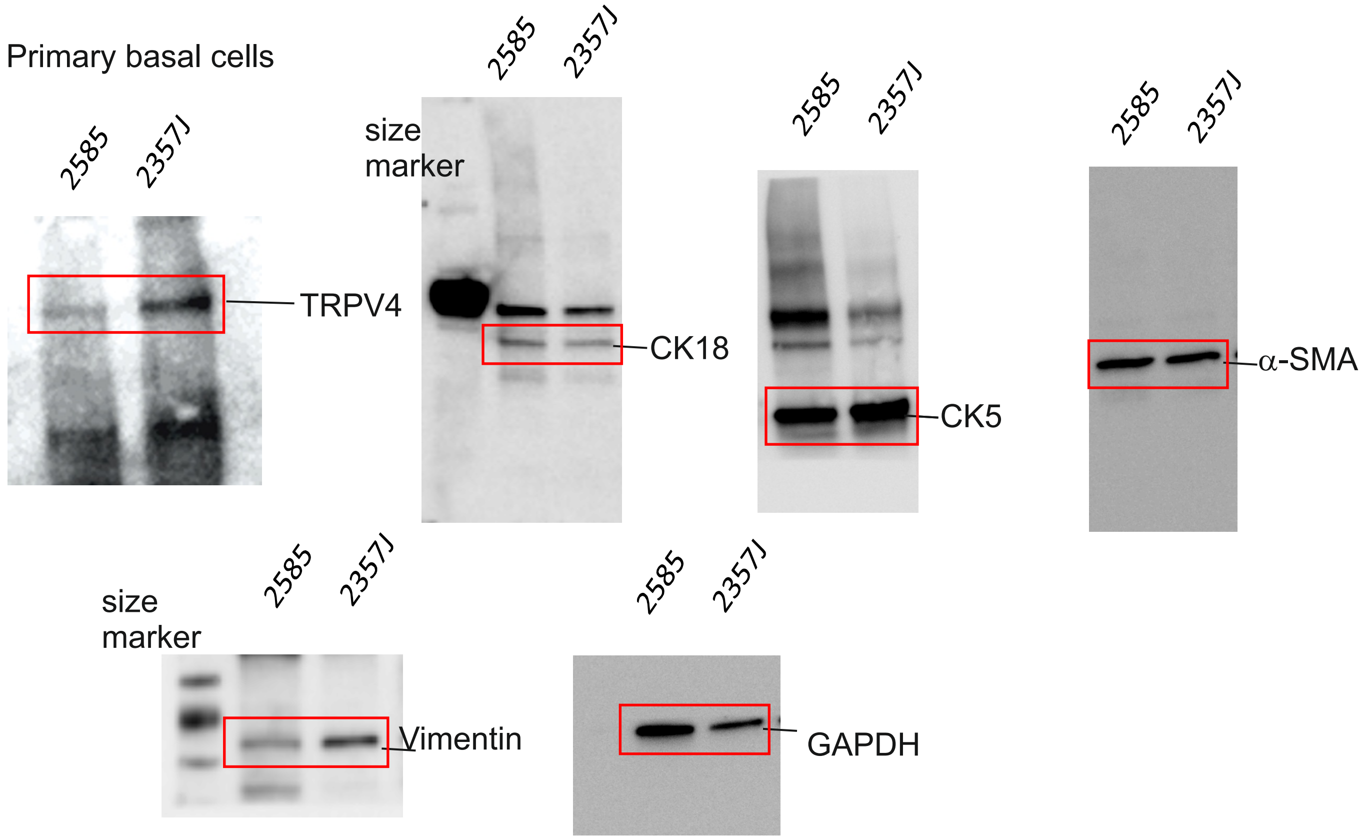

Uncropped/unmodified Western Blots

Related to Figure 2A, 2E, 2I and S2A and S2C

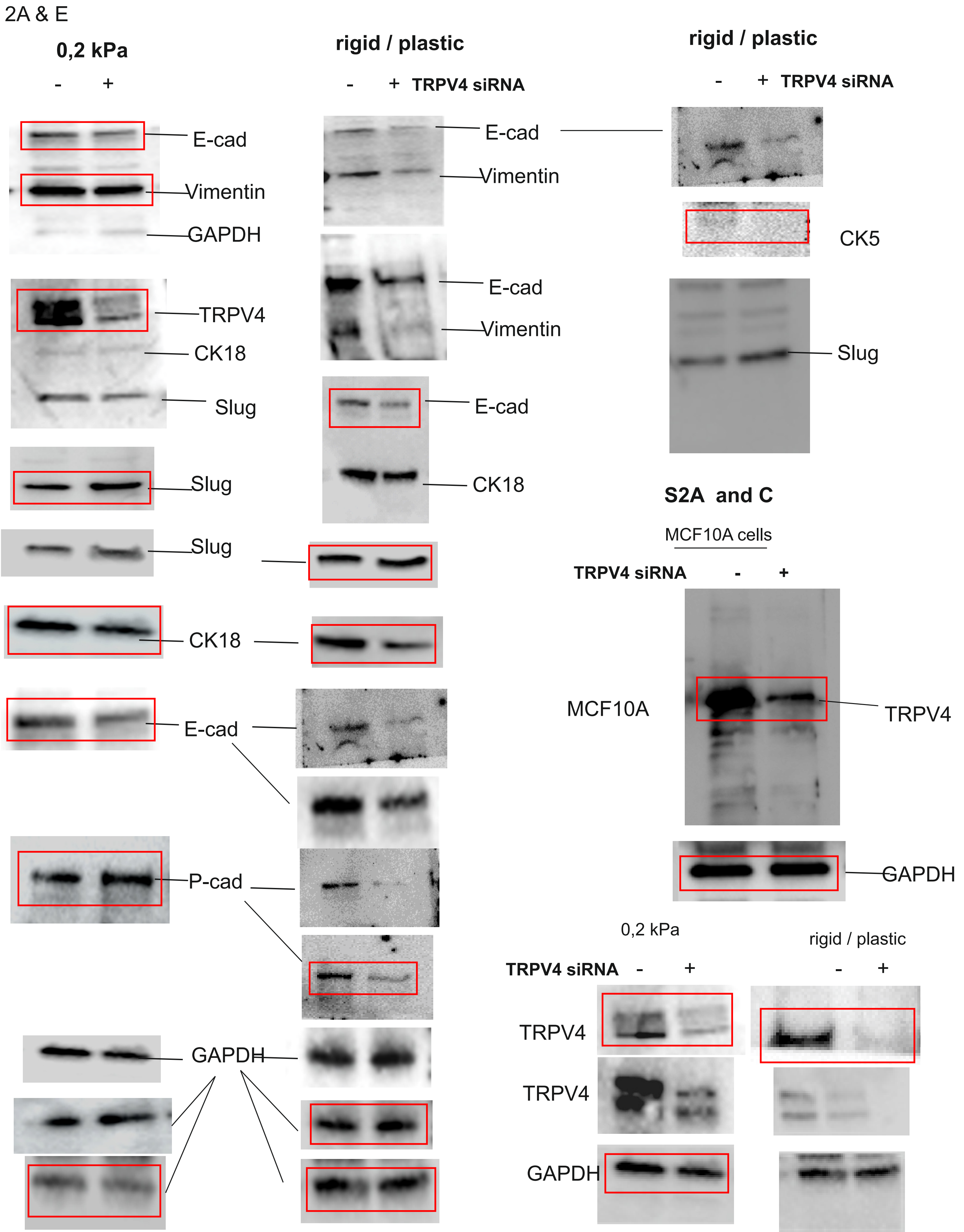

Uncropped/unmodified Western Blots

Related to Figure S3A and S6C

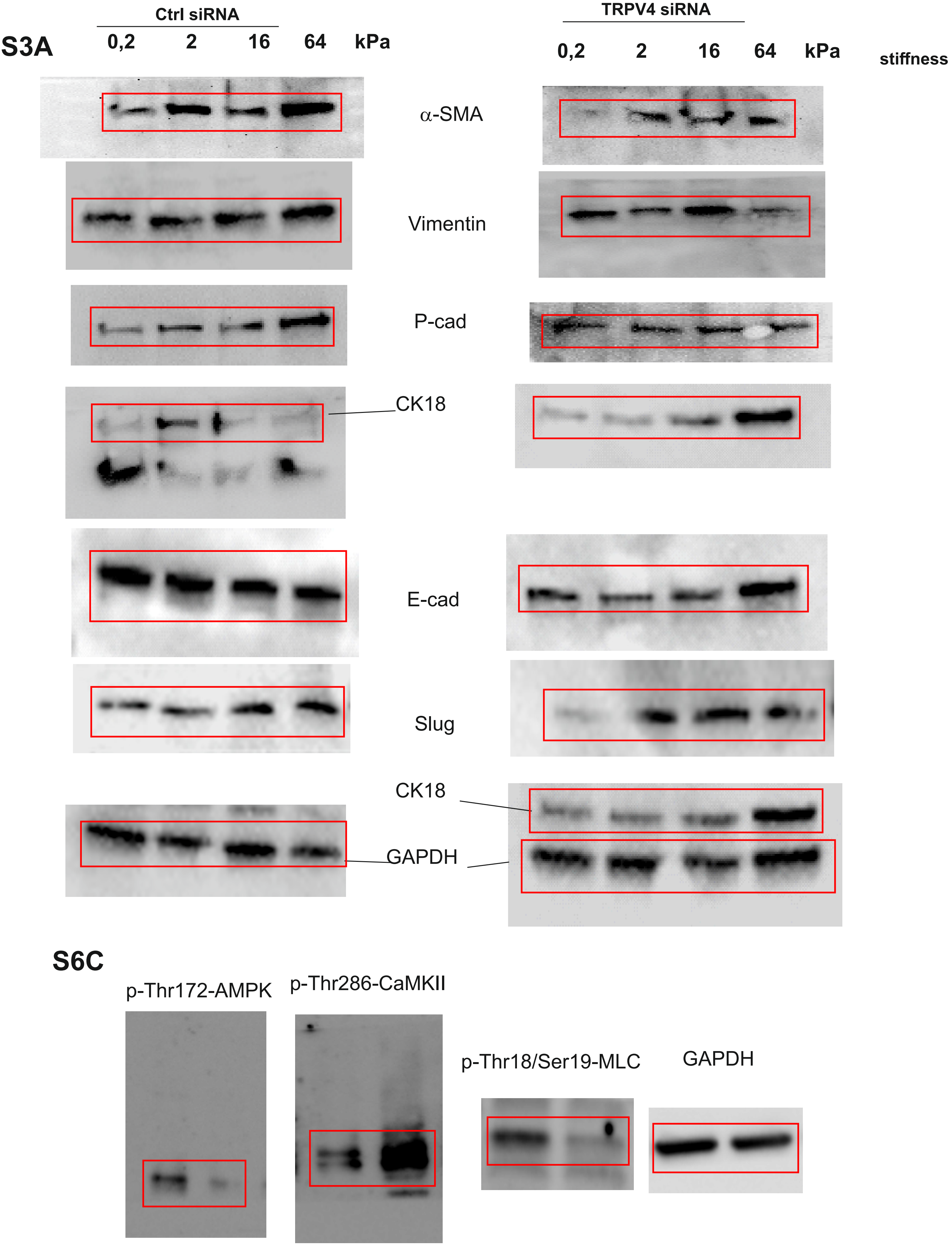

Supplement: Supplementary file 2 — Supplementary Information 2. [file 41598_2024_57346_MOESM2_ESM.pdf]
